# Spotlight on pyroptosis: role in pathogenesis and therapeutic potential of ocular diseases

**DOI:** 10.1186/s12974-022-02547-2

**Published:** 2022-07-14

**Authors:** Meini Chen, Rong Rong, Xiaobo Xia

**Affiliations:** 1 Eye Center of Xiangya Hospital, Central South University, Changsha, 410008 Hunan People’s Republic of China; 2 Hunan Key Laboratory of Ophthalmology, Changsha, 410008 Hunan People’s Republic of China; 3 National Clinical Research Center for Geriatric Diseases (Xiangya Hospital), Changsha, 410008 Hunan People’s Republic of China

**Keywords:** Caspase-1, Caspase-4/5/11, Eye, Gasdermin, Inflammasome, Pyroptosis

## Abstract

Pyroptosis is a programmed cell death characterized by swift plasma membrane disruption and subsequent release of cellular contents and pro-inflammatory mediators (cytokines), including IL‐1β and IL‐18. It differs from other types of programmed cell death such as apoptosis, autophagy, necroptosis, ferroptosis, and NETosis in terms of its morphology and mechanism. As a recently discovered form of cell death, pyroptosis has been demonstrated to be involved in the progression of multiple diseases. Recent studies have also suggested that pyroptosis is linked to various ocular diseases. In this review, we systematically summarized and discussed recent scientific discoveries of the involvement of pyroptosis in common ocular diseases, including diabetic retinopathy, age-related macular degeneration, AIDS-related human cytomegalovirus retinitis, glaucoma, dry eye disease, keratitis, uveitis, and cataract. We also organized new and emerging evidence suggesting that pyroptosis signaling pathways may be potential therapeutic targets in ocular diseases, hoping to provide a summary of overall intervention strategies and relevant multi-dimensional evaluations for various ocular diseases, as well as offer valuable ideas for further research and development from the perspective of pyroptosis.

## Background

Programmed cell death (PCD) plays an important role in regulating stress response, homeostasis, and diseases. PCD was first identified in 1972 when the ultrastructural features of cells undergoing apoptosis were revealed [1]. With extensive research, new observations of PCD are rapidly emerging, both refining and redefining known patterns of cell death, such as apoptosis, and unmasking previously undescribed forms of cell death, such as necroptosis, autophagy, ferroptosis, and pyroptosis [2]. Recently, pyroptosis has received increased attention in the pathogenesis of various diseases [3–5]. Pyroptosis is an inflammatory form of cell death executed by gasdermins, a family of transmembrane pore-forming proteins activated via inflammasome-dependent or inflammasome-independent pathways [3]. It was first identified in macrophages infected with *Shigella flexneri* in 1992 and was named in 2001 [6, 7]. Pyroptosis also participates in the innate immune response by inhibiting intracellular pathogen replication and inducing pathogen elimination by the immune cells. When pyroptosis is uncontrolled, inflammatory responses in neighboring cells and tissues are activated, further exacerbating the inflammatory damage [8].

Importantly, the innate immune system maintains and controls eye homeostasis and visual function by acting as an early responder to environmental perturbations with the assistance of the blood–retinal barrier, which is composed of and regulated by highly specialized cell types [9]. The balance between cell growth and death in eye tissues and the optic nerve contributes to homeostasis. Previous studies have agreed that the progression and prognosis of ocular diseases are attributed to apoptosis and necrosis. However, with the development of biotechnology and intensive studies on cell death, researchers have discovered that ocular cells engaged in the progression of ocular diseases not only typically undergo apoptosis and necrosis, but also show characteristics of other forms of cell death, including pyroptosis. Emerging evidence shows that pyroptosis is tightly linked to the initiation and progression of ocular diseases, including age-related macular degeneration (AMD), glaucoma, diabetic retinopathy (DR), AIDS-related human cytomegalovirus (HCMV) retinitis, dry eye disease (DED), keratitis, uveitis, and cataract [10–15]. At the cellular level, retinal ganglion cells (RGCs), retinal pigment epithelial (RPE) cells, retinal pericytes, trabecular meshwork cells, corneal epithelial cells (CECs), lens epithelial cells (LECs), retinal microvascular endothelial cells (RMECs), Müller cells, astrocytes, and microglia are involved in pyroptosis in various ocular diseases [16–23]. This review provides a systematic overview of the evidence for pyroptosis and its functional role in ocular diseases, emphasizing the molecular signaling pathways involved in the vision system. In addition, we briefly summarize the connectivity and differences between pyroptosis and other forms of PCD that contribute to ocular diseases, as well as discuss the therapeutic potential of pyroptosis in ocular diseases.

## Morphological and biological characteristics of pyroptosis

Pyroptosis is a type of lytic cell death characterized by swift plasma membrane disruption and subsequent release of cellular contents and pro-inflammatory mediators, including IL‐1β and IL‐18 [5]. It occurs primarily in phagocytes of the myeloid lineage, such as macrophages, dendritic cells, and neutrophils, as well as in CD4+ T cells, keratinocytes, epithelial cells, endothelial cells, and neurons [24]. Focusing on eye structure, RGCs, Müller cells, astrocytes, microglia, endothelial cells, pericytes, retinal epithelial cells, and CECs are the main ocular linked to pyroptosis [16, 19, 25]. Inflammatory caspases that cleave pro-IL‐1β and pro-IL‐18 into their mature form and drive pyroptosis (i.e., caspases 1 and 11 in mice, and caspases 1, 4, and 5 [the latter two are human homologues of caspase 11] in humans) are highly expressed in these cells [24, 26].

Pyroptosis has some distinct characteristics compared to other forms of PCD (Table 1). First, the nuclei of pyroptotic cells undergoing chromatin condensation and DNA fragmentation remain intact, whereas their plasma membranes are disrupted [27]. Ultrastructural studies of ARPE-19 cells and retinal stem cells showed cytoplasmic swelling, mitochondrial damage, and autophagosome-like structures [28, 29]. In other forms of PCD such as apoptosis, when they undergo DNA damage, in contrast to pyroptotic cells, the nucleus is damaged and the plasma membrane remains intact [27]. In cells undergoing necroptosis and ferroptosis, high-mobility group box 1 (HMGB1) can pass through ruptured nuclear and cytoplasmic membranes. Furthermore, nuclear membrane damage is observed prior to cytoplasmic membrane rupture during ferroptosis [30]. Notably, although cells undergoing NETosis do not suffer from chromatin condensation, their nuclear and plasma membranes both rupture, leading to neutrophil cell lysis [31]. Moreover, pore formation induced by inflammation leads to osmotic swelling and cell lysis in pyroptotic cells [27]. Activation of caspase-1/4/5/11 and cleavage of gasdermin D (GSDMD) contribute to the transmembrane pore formation of these cells, and the open GSDMD pore breaches the normal permeability barrier of the plasma membrane [27, 32]. The abnormal separation of sodium and potassium on both sides of the cell membrane results in a massive sodium influx, which brings with hydrating water. Subsequently, cell volume begins to increase, resulting in cell swelling and osmotic lysis [27, 32]. Compared to pyroptotic cells, apoptotic cells maintain intact membranes [33]. In necroptosis and ferroptosis, although cell swelling and osmotic lysis are caused by an abnormal permeability barrier of the plasma membrane, unlike pyroptosis, the process does not depend on caspase-1/4/5/11 and GSDMD but on other effectors, such as RIPK1‐RIPK3‐MLKL and PUFAs, respectively. Additionally, two recent reports demonstrated that GSDMD regulates cell death via NETosis [34]. Previous research also claimed that the caspase family mediates both apoptosis and pyroptosis, although caspases 3, 6, 7, and 8 were once thought to be distinct markers of apoptosis [27]. However, a new mechanism of caspase-3/8-mediated pyroptosis has recently been reported [8, 35–39].

**Table 1:** Comparison of different forms of cell death and their biological characteristics

	Pyroptosis	Apoptosis	Necroptosis	Ferroptosis	NETosis	Autophagy
Death stimulus	DAMPs and PAMPs, dsDNA, pathogens, LPS, anthrax lethal toxin, microbial toxins and metabolites, viral RNA, extracellular ATP, lysosomal damage, permeabilization of the cell membrane to potassium ions, etc.	DNA damage, hypoxia, viral infection, toxins	Ischemia–reperfusion, physical or chemical trauma, viral or bacterial infection, etc.	Decreased cysteine (e.g., erasin) or glutamine uptake, increased iron uptake (extrinsic), inhibition of GPX4 (e.g., RSL3) (intrinsic)	Bacterial components, fungal β-glucan, cytokines	mTOR inhibitor, trehalose, treatment with etoposide, staurosporine, thapsigargin
Initiator	Activation of inflammasomes (e.g., NLRP3, NLRC4, AIM2, pyrin, etc.)/DR binding to TAK1/activation of GzmA or GzmB/neutrophil elastase/cathepsin G	Death receptor (TNF superfamily) activation (extrinsic)/ intracellular signals (intrinsic)	Death receptor activation (TNFR1, CD95, TRAIL-R1, TRAIL-R2, TLR3, TLR4, ZBP1) dependent on caspase-8 inhibition	System $\chi_{{\text{c}}}^{ - }$ inhibition, depletion of GSH	Activation of neutrophil surface receptors, such as GPCRs, TNF, and Fc receptors (neutrophil activation)	ULK complex (consisting of ATG101, ATG13, FIP200) and ATG9
Regulator/mediator	Nek7	Caspase-8 (extrinsic)/caspase-9 (intrinsic)	RIPK1‐RIPK3	Inhibition of GPX4, iron metabolism-related proteins	Combined action of PAD4, NE, and MPO, serine proteases	PtdIns3K complexes, ATG2-Atg18/WIPI complexes, the ATG12 conjugation system, the Atg8/microtubule-associated protein 1 light chain 3 (LC3) conjugation system
Executor	Caspase‐1, caspase‐4/5/11, caspase-3/8, gasdermin family	Caspase‐3, 7	MLKL oligomerization, translocation to the inner leaflet of the plasma membrane	Iron-dependent lipid peroxidation	Dysregulated NETs, GSDMD	Autophagosome, autolysosome
Morphology						
Plasma membrane rupture	Yes	No (membrane blebbing)	Yes	Yes	Yes	No
Cell swelling	Yes	No (cell shrinkage)	Yes	No (rounding up)	Yes	No
Nuclear membrane integrity	Yes	No	No	No	No	–
DNA fragmentation	Yes	Yes	Yes	No	No	–
Chromatin condensation	Yes	Yes	Yes	No	No	–
Mitochondria damage	Yes (swelling)	Yes (swelling?/condensation?)	Yes (ruptured mitochondrial outer membrane, decreased or vanished mitochondria cristae, condensed mitochondrial membrane)	Yes (membrane densities, reduction or vanishing of mitochondria crista, outer mitochondrial membrane rupture)	Yes (swelling)	Yes
Lytic cell death	Yes	No	Yes	Yes	Yes	–
Inflammation	Yes	No	Yes	Yes	Yes	Partially have

*DAMP* danger-associated molecular pattern, *PAMP* pathogen-associated molecular pattern, *dsDNA* double-stranded DNA, *LPS* lipopolysaccharide, *NLRP* NOD-like receptor protein, *AIM2* absent in melanoma 2, *TAK1* TGF-β-activated kinase-1, *Gzm* granzyme, *TNF* tumor necrosis factor, *TRAIL-R* TNF-related apoptosis-inducing ligand receptor, *TLR* Toll-like receptor, *ZBP1* Z-DNA binding protein 1, *RIPK* receptor interacting serine/threonine kinase, *TRADD* tumor necrosis factor receptor type 1-associated death domain protein, *MLKL* mixed lineage kinase domain-like, *GPX* glutathione peroxidase, *GSH* glutathione, *GPCR* G-protein-coupled receptors, *PAD* peptidyl arginine deiminase, *MPO* myeloperoxidase, *NE* neutrophil elastase, *NET* neutrophil extracellular trap, *GSDMD* gasdermin D, *mTOR* mammalian target of rapamycin, *ULK* Unc-51-like kinase, *PtdIns3K* class III phosphatidylinositol 3-kinase, *WIPI* WD repeat domain phosphoinositide-interacting protein

### Inflammasomes

Inflammasomes are cytosolic immune signaling complexes that cause inflammation and pyroptosis [40]. They assemble in response to pathogen- or damage -associated molecular patterns (PAMPs or DAMPs). An inflammasome typically comprises a sensor, an adaptor, and zymogen procaspase-1 [41]. Inflammasome-initiating sensors are pattern recognition receptors (PRRs), including nucleotide-binding oligomerization domain (NOD)- and leucine-rich repeat (LRR)-containing receptors (NLRs), absent in melanoma-2 (AIM2)-like receptors (ALRs), and proteins with a tripartite motif (TRIM) such as pyrin [41, 42]. Upon detection of specific stimuli, sensors recruit the inflammasome adaptor apoptosis-associated speck-like protein containing a caspase activation and recruitment domain (CARD) (ASC) to form a multimeric complex called ‘speck’ [41]. The CARD of ASC allows ASC to couple the upstream sensor PRRs to the effector cysteine protease caspase-1 and then convert the effector into its bioactive form [40, 41]. Notably, the CARD of NLRC4 and murine NLRP1b, members of the NLR family, can interact directly with caspase-1 without the help of ASC [40]. Active caspase-1 triggers pyroptosis via the canonical pathway, whereas activated caspase-4/5/11 causes pyroptosis via the noncanonical pathway [27]. These inflammasomes have been shown to participate in the pathogenesis of various ocular diseases.

#### NLRP3 inflammasome

The NOD-like receptor protein 3 (NLRP3) of the NLR family is the most well-investigated inflammasome sensor. NLRP3 consists of three parts: an amino-terminal PYRIN domain (PYD), a nucleotide-binding domain (NACHT), and a carboxy­terminal LRR domain [43]. Because of its ability to respond to various stimuli, NLRP3 is a critical integrator of cellular stress. These stimuli, including, but not limited to, pathogens, microbial toxins, viral RNA, extracellular ATP, lysosomal damage, and permeabilization of the cell membrane to potassium ions, might lead to the loss of autoinhibition of NLRP3 [40, 43]. NLRP3 is expressed in most ocular tissues and cells and is upregulated in multiple ocular diseases.[44, 45].

NIMA-related kinase 7 (Nek7) is an important component of the murine NLRP3 inflammasome that binds to the LRR and NACHT of NLRP3 [40]. Nek7 is necessary for NLRP3 inflammasome activation, but dispensable for NLRC4 and AIM2 inflammasome activation. In addition, Nek7 could control NLRP3 oligomerization, ASC speck formation, and caspase-1 activation [46]. However, the presence of Nek7 is insufficient to drive NLRP3 activation; priming and activation are the two main steps required for complete activation of NLRP3 [40]. The priming step licenses the cell, whereas the activation step induces full activation and inflammasome formation [46].

The priming step has two major functions [47]. First, it upregulates NLRP3 by promoting transcription [40, 43]. After recognizing PAMPs or DAMPs by PRRs, the transcription factor nuclear factor-κB (NF-κB) is activated and induces the transcription of NLRP3, as well as pro‑IL‑1β and pro-IL-18 [43]. With the help of several mediators that promote signal transduction of PRRs, such as myeloid differentiation primary response 88 (MyD88) and Fas-associated protein with death domain (FADD), the cell is ‘primed’ and ready for the next step [40]. Aside from upregulating NLRP3, priming induces post-translational modifications of NLRP3, including ubiquitination, phosphorylation, and SUMOylation. Modified NLRP3 is maintained in an auto-inhibited inactive but signal-competent state [40, 43, 47].

The second step occurs after recognizing the NLRP3 activator [47]. Several NLRP3 activation models, which may act in tandem or independently, are now widely accepted, including pore formation and ion redistribution, lysosomal disturbance, metabolic dysfunction, mitochondrial dysfunction, Golgi apparatus disassembly, and noncanonical NLRP3 activation models [43, 48]. When a primed cell is subjected to an activating stimulus under appropriate conditions, NLRP3 starts to activate and assemble [43, 49]. In AMD, complement components, amyloid-beta (Aβ), and by-products of oxidation, which are rich in drusen composition as well as lysosomal destabilization, release of cathepsins, and Alu RNA, are considered as relevant NLRP3 activators [50]. In DR, multiple diabetes mellitus-related metabolic factors such as ATP, cholesterol levels, and cellular structure destabilization, including mitochondrial dysfunction, lysosomal rupture, as well as molecular or ion perturbation such as ROS, K^+^ efflux, and Ca^2+^ signaling, are a second signal for NLRP3 activation and assembly [51].

Interestingly, there are some exceptions to this two-step activation mode of the NLRP3 inflammasome. First, TLR4 stimulation alone can solely induce IL-1β secretion in human and porcine monocytes [47]. Second, in mouse bone marrow-derived and splenic dendritic cells, a single lipopolysaccharide (LPS) stimulus is sufficient to activate the NLRP3 inflammasome [47, 52]. Another exception is that the stimulation of TLRs, together with extracellular ATP, rapidly activates NLRP3 in mouse bone marrow-derived macrophages [47].

#### AIM2 inflammasome

The AIM2 inflammasome has received the most attention in terms of infections. Its activation by sensing double-stranded DNA (dsDNA) from viruses, bacteria, and parasites, as well as host-self DNA, causes cytokine production and GSDMD-mediated pyroptosis [53]. AIM2 is a cytosolic receptor with two major domains: a PYD domain and HIN-200 domain [5, 40]. Under homeostatic conditions, the HIN domain functions as a suppressor of the PYD domain, causing the latter to fail to recruit ASC. Once dsDNA from the pathogen is sensed by the HIN domain, the PYD domain is displaced from the HIN domain and can accept the PYD domain of ASC [54, 55]. The activated AIM2 inflammasome then generates bioactive caspase-1 in an autocatalytic manner [55]. Although AIM2 has been reported in various diseases, little is known about its role in the development of ocular diseases. In fact, a study showed that AIM2−/− mice did not show improved retinal function or survival as retinal degenerative disorders progressed, while NLRP3−/− mice showed a protective effect [56]. Therefore, the contribution of AIM2-mediated pyroptosis to the pathogenesis of ocular diseases remains unclear.

#### Pyrin inflammasome

Pyrin (also known as marenostrin, TRIM 20) is a protein encoded by the *MEFV* (human) or *Mefv* (mouse) gene that is expressed mainly in neutrophils, monocytes, and dendritic cells [57, 58]. Under homeostasis, pyrin senses the modifications of the small GTPase RhoA activity. RhoA activation leads to pyrin inhibition [57]. When the inactivation of Rho GTPases by effector proteins or pathogenic toxins is recognized by pyrin, pyrin-dependent pyroptosis is induced [40, 58]. Unfortunately, to the best of our knowledge, the pyrin signaling pathway has not been explored in ocular diseases.

#### NLRC4

NLRC4 inflammasome activation is triggered by flagellin and type III secretion systems (T3SS) apparatus, both of which are bacterial components [58]. Neuronal apoptosis inhibitory proteins (NAIPs) are required for NLRC4 activation to recognize the presence of these two bacterial components in the cytosol [58]. NAIP alters conformation upon ligand engagement, allowing oligomerization with NLRC4 [59]. NLRC4 can directly activate CASP1, which cleaves GSDMD and IL-1β/18 and initiate pyroptosis [59]. NLRC4 is also expressed in the cornea and retina of the eye [44]. Furthermore, NLRC4 has been demonstrated to promote pyroptosis in DED and acute glaucoma [11, 12].

#### NLRP1

Human NLRP1 was the first identified inflammasome sensor shown to play a role in pyroptosis initiation. Both human and mouse NLRP1 lack a PYD, with the latter carrying three NLRP1 paralogs (NLRP1a–c) [40]. Several activators of NLRP1 have been identified, including anthrax lethal toxin deployed by *Bacillus anthracis*, bacterial muramyl dipeptide, *Toxoplasma gondii*, and the DPP8/9 inhibitor Val‐boroPro [60, 61]. In addition, NLRP1 can detect metabolic disturbances [60]. Reduced cellular ATP levels are thought to be correlated with NLRP1 inflammasome activation [61]. NLRP1 appears in the cornea and retina of the eye [44]. Previous studies have shown that targeting NLRP1 can attenuate pyroptosis and the progression of acute glaucoma and DR [22, 62, 63]. Moreover, the NLRP1 inflammasome is activated by CASP8 in acute glaucoma [64].

#### Other forms of inflammasomes

Apart from these inflammasomes, other inflammasomes have been reported to be related to pyroptosis in ocular diseases. In mice, NLRP6 is a negative regulator of NF-κB and MAPK signaling in response to Gram-positive and Gram-negative bacteria [40]. It is found mostly in corneal epithelial and conjunctival epithelial cells [44]. Furthermore, the NLRP3/6 inflammasome balance is regulated by lncRNA-H19, which functions with miR-21 and PDCD4. Changes in this balance stimulate pyroptosis of microglia and neuronal death in the retina [65]. Moreover, the collaboration between NLRP12 and NLRC4 has been reported to initiate GSDMD-dependent pyroptosis in the mucosal corneal epithelium [12]. The regulatory function of NLRC5 in microglial pyroptosis also relies on its cooperation with NLRP3 and NLRC4 in high intraocular pressure (IOP)-induced ischemic retinopathy. Neurotoxic factors secreted by microglia in response to pyroptosis further harm RGCs and cause irreversible damage [66].

### Gasdermin family

The GSDM family is an executor of pyroptosis and includes GSDMA, GSDMB, GSDMC, GSDMD, GSDME (DNFA5), and PVJK (also known as GSDMF/DFNB59), with GSDMB being regarded as a unique gene in the human genome. Every GSDM member is expressed distinctly and restrictedly in different tissues, leading to various diseases [67]. Except for DFNB59, all members of the GSDM family have been reported to be involved in pyroptosis [68]. Nevertheless, only GSDMD and GSDME have been investigated for the pathogenesis of ocular diseases. GSDMD is the best-known member of the GSDM family and plays a central role in pore formation and subsequent pyroptosis. GSDMD has also been implicated in several ocular diseases. As for GSDME, Liao et al. showed that abnormal accumulation of all-trans retinal (atRAL) was associated with caspase-3/GSDME-mediated pyroptosis of RPE cells and photoreceptors [69, 70]. However, we could not find any other evidence regarding ocular diseases related to GSDME-mediated pyroptosis. Recent studies on GSDME were mainly reported in cancer cells, showing that GSDME could induce pyroptosis and cause cancer cell death [36].

GSDM consists of an N-terminal pore-forming domain (PFD), a linker, and a C-terminal repressor domain [3]. Generally, PFD activity can be inhibited by the repressor domain. When stimulated by intrinsic or extrinsic factors, gasdermins are cleaved by caspases, granzymes (GZMs), neutrophil elastase, or some cathepsins within the linker region, and free the PFD to oligomerize and form pores in the cell membrane. As a result, inflammatory cytokines release and pyroptosis occurs [71]. More specifically, GSDMD is cleaved by caspase-1/4/5/11 [72] as well as neutrophil elastase, cathepsin G, and CASP8 [39, 73, 74]. Meanwhile, GSDME is cleaved by GZMB and CASP3 [37, 68], GSDMB is cleaved by GZMA, and GSDMC is cleaved by CASP8 [75, 76]. The exact execution of GSDMA cleavage remains unknown for a long time. Recently, a 2022 novel study revealed that streptococcal pyrogenic exotoxin B (SpeB), a *Streptococcus* cysteine protease, cleaves GSDMA and triggers pyroptosis [77]. We believe that more studies will be conducted to determine additional GSDM cleavage executors. To understand the underlying mechanism, the exact cleavage sites of each GSDM and their changes in different species, tissues, or cells require further investigation.

Notably, it has been proven that GSDME-N mechanistically links the activation of inflammasome-mediated pyroptosis to apoptosis. First, GSDME-N enhances the activation of the apoptotic protease activating factor 1 (Apaf-1) apoptosome by permeabilizing mitochondria and releasing cytochrome c (cyt c), providing a positive feedback on caspase-3 activation and GSDME cleavage. The GSDME-N pores on the plasma membrane cause pyroptosis, allowing the release of DAMPs such as HMGB1. GSDMA and GSDMD also have this mitochondrial pore-forming function; however, the feedback loop remains unknown [78].

## Molecular mechanisms of pyroptosis in ocular diseases

**Fig. 1:**
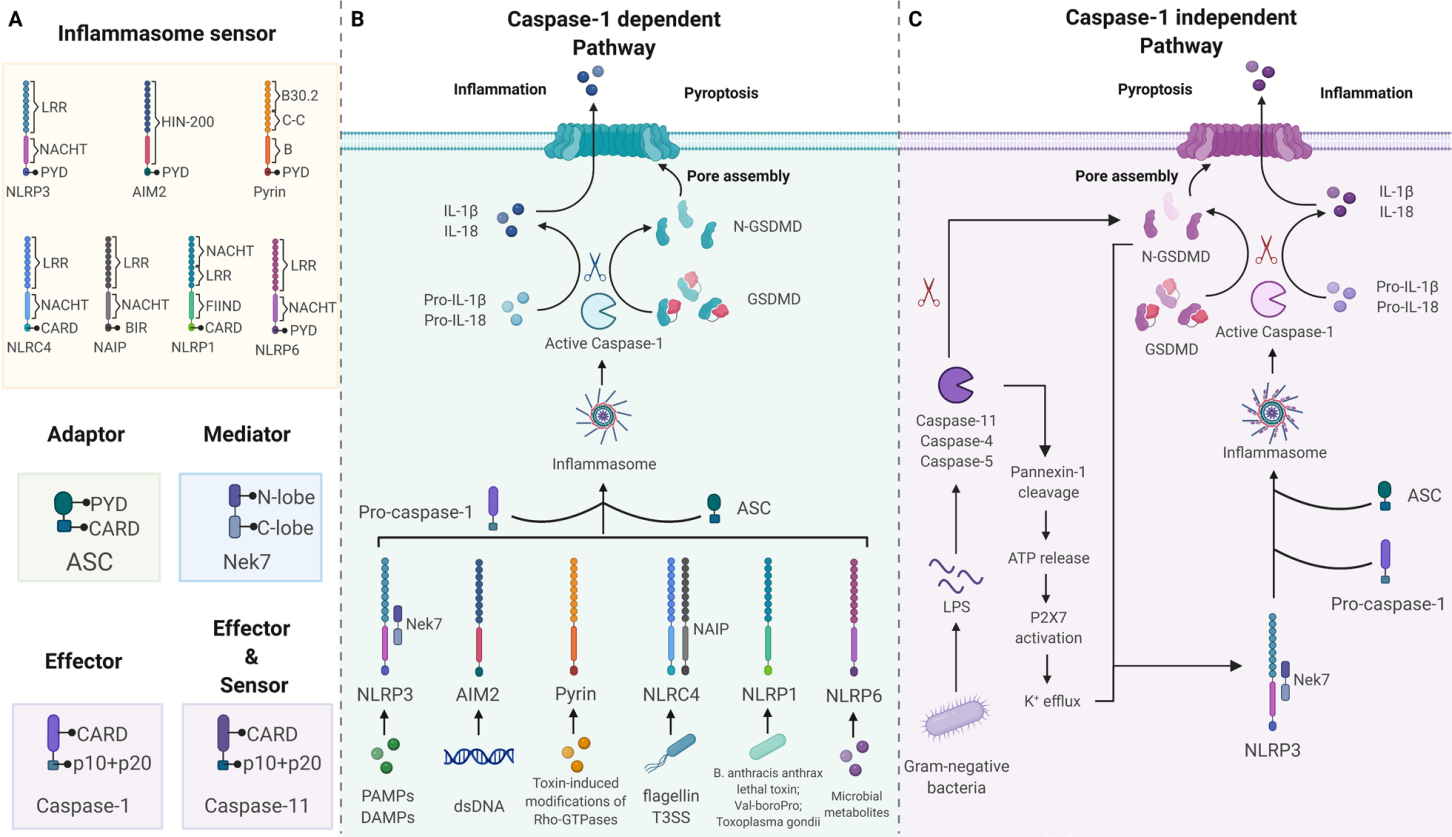
Inflammasome-dependent pathway of pyroptosis. **A** Inflammasome sensors are cytosolic proteins that contain a PYD and/or a CARD. They may also contain a LRR, NACHT, HIN-200 domain, B30.2 domain, C–C, B-box domain (B), BIR, or FIIND. Upon detection of specific stimuli, sensors with a PYD recruit adaptor protein ASC to mediate CARD–CARD interactions with the effector cysteine protease caspase-1. Of note, NLRC4 and murine NLRP1b can interact directly with caspase-1 without ASC recruiting them. Nek7 is an important component of the murine NLRP3 inflammasome, binding to the LRR and NACHT of NLRP3. **B** Canonical pathway: inflammasome sensors can be activated by various signals followed by oligomerization with ASC and pro-caspase-1. Activated caspase-1 cleaves GSDMD to release the N-terminal domain (GSDMD-N), which then induces pyroptosis. Caspase-1 also cleaves pro-IL-1β and pro-IL-18 into their active forms, which are released through GSDMD pores. **C** Noncanonical pathway: caspase-4/5/11 can be directly activated by LPS, leading to GSDMD cleavage and cell contents and K^+^ release. K^+^ efflux further promotes the activation of caspase-1. *PYD* pyrin domain, *CARD* caspase activation and recruitment domain, *LRR* leucine-rich repeat domain, *NACHT* nucleotide-binding NACHT domain, *C–C* coiled–coil domain, *BIR* baculovirus inhibitor of apoptosis repeat, *FIIND* function-to-find domain, *Nek7* NIMA-related kinase 7, *GSDMD* gasdermin D, *IL* interleukin, *LPS* lipopolysaccharide

**Fig. 2:**
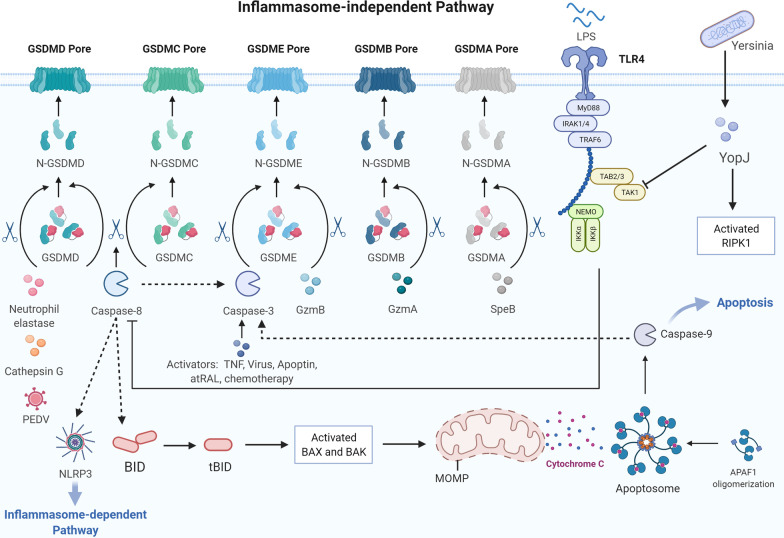
Inflammasome-independent pathway of pyroptosis. YopJ expressed during Yersinia infection inhibits TGF-β-activated kinase 1 (TAK1) and induces caspase-8-related cleavage of GSDMD and GSDMC. Neutrophil elastase, porcine epidemic diarrhea virus, and cathepsin G cleave GSDMD. Caspase-8 acts upstream of caspase-3 through the Bax/Bak signaling, while caspase-3 mediates GSDME cleavage. GZMA and GZMB activate GSDMB and GSDME, respectively. Streptococcal pyrogenic exotoxin B (SpeB) could cleave GSDMA and trigger pyroptosis

GSDM-induced pyroptosis involves both inflammasome-dependent and inflammasome-independent pathways. Generally, inflammasome-dependent pyroptosis (Fig. 1) includes the CASP1-dependent pathway (canonical pathway) and CASP4/5/11-dependent pathway (noncanonical pathway). Recent studies have revealed new inflammasome-independent pathways (Fig. 2), including the caspase-3/8-mediated pathway, and other GSDM-mediated pathways.

### Inflammasome-dependent pathway

#### Canonical pathway

In the canonical pyroptosis pathway, the formation of a functional inflammasome starts with PRRs, including NLRs, AIM2, or pyrin, by sensing PAMPs and DAMPs under the stimulation of hyperlipidemia, hyperglycemia, and inflammation [5, 40]. PRRs that recognize the pathogenic stimuli then bind to pro-CASP1 with the assistance of ASC speck and eventually form inflammasomes [8]. Upon inflammasome formation, CASP1 is activated. Active CASP1 then cleaves the pyroptosis executor GSDMD and unmasks the pore-forming domain from GSDMD-C inhibition [72, 79]. Active CASP1 also proteolytically matures pro-IL-1β and pro-IL-18 into their active forms [24]. The liberating GSDMD-N, which is recruited to the inner side of the cell membrane, oligomerizes to form a transmembrane pore. Transmembrane pores result in K^+^ efflux and water influx, causing cell swelling, cell rupture, and release of cellular contents including bioactive IL-1β and IL-18, resulting in a further magnified inflammatory response in the host [40, 72, 79].

#### Noncanonical pathway

In the noncanonical pathway, LPS, a component of the Gram‐negative bacterial cell wall, is recognized by CASP4/5/11 in the host. Subsequently, activated CASP4/5/11 initiates GSDMD cleavage and pyroptosis [5, 27, 80, 81]. Although CASP11 can cleave GSDMD, it is unable to transform pro-IL-1β and pro-IL-18 into their bioactive forms [26]. Therefore, the noncanonical pathway still requires caspase‐1 for its indispensable role in producing mature IL‐1β and IL‐18 [5, 80]. Additionally, activated CASP11 also cleaves Pannexin-1, through which ATP outflows to the extracellular space, binds to P2X7R, and triggers NLRP3-related pyroptosis [71].

### Inflammasome-independent pathway

#### Caspase-3/8-mediated pathway

CASP3/8 is traditionally considered a distinct marker and molecule of apoptosis; however, recent studies have shown that they are also important executors in pyroptosis. Instead of GSDMD, CASP3 cleaves GSDME in response to activators such as chemotherapeutic agents, TNF, viral infection, apoptin, and atRAL in the RPE [32, 36, 69, 82–84]. CASP3 is often activated after CASP8 or CASP9 activation; thus, CASP8 is an upstream activator of CASP3 and is known to control apoptotic cell death. Previous studies have also shown that CASP8 plays different roles in pyroptosis. First, it directly cleaves GSDMD to induce pyroptosis [85]. Researchers found that the Yersinia effector protein YopJ inhibits TGF-β-activated kinase-1 (TAK1) or IKK kinases and results in CASP8-dependent cleavage of GSDMD [86]. Second, CASP8 can affect the priming, activation, and post-assembly steps of the NLRP3 inflammasome [87]. Third, CASP8 acts upstream of CASP3 to regulate pyroptosis [85]. Apart from directly cleaving pro-CASP3, CASP8 also cleaves Bid, which belongs to the B-cell lymphoma 2 (Bcl-2) family, into its shortened form ‘tBid’. tBid migrates to the mitochondria, where it generates Bax/Bak pores on the surface (mitochondrial outer membrane pore [MOMP]), releases cyt c, and activates CASP3 and CASP9 [82, 88, 89]. The assembly of the Apaf-1/CASP9 apoptosome promoted by the release of cyt c contributes to the CASP3/GSDME pathway [89]. Nevertheless, to the best of our knowledge, there is very little research on the CASP3/GSDME pathway conducted in the ophthalmology field, suggesting that extensive research on this pathway can be conducted in ocular diseases in the future. Fourth, CASP8 matures pro-IL-1β into its bioactive form in a noncanonical manner. Currently, we know that IL-1β, the key cytokine involved in pyroptosis, is processed and secreted with the help of CASP1. However, in the inflammasome-independent pathway, we noticed that there was little involvement of CASP1 in the whole process, unless CASP8 could activate the NLRP3 inflammasome and link it to CASP1-dependent cytokine release. In fact, IL-1β still engages in inflammasome-independent pyroptosis without the assistance of CASP1. Scientists have shown that, in the absence of CASP1, noncanonical maturation of IL-1β is conducted by CASP8 under various stimulations [90, 91]. However, this noncanonical processing is not as efficient as canonical processing. Therefore, in the future, a comparison of the efficiency of each pyroptotic pathway might be a potential issue. Furthermore, the underlying mechanism of the noncanonical maturation of IL-1β in inflammasome-independent pyroptosis or even in the entire immune system is worth further investigation. Finally, CASP8 cleaves GSDMC into GSDMC-N, generating pores on the cancer cell membrane to prompt pyroptosis under the stimulation of TNF-α treatment or the metabolite α-ketoglutarate treatment [76, 92]. Currently, there has been no research on the CASP8/GSDMC pathway in the ophthalmology field.

#### Other GSDM-mediated pathways

More recently, researchers have reported that GZMA from cytotoxic lymphocytes cleaves GSDMB and GZMB from natural killer cells, and cytotoxic T lymphocytes cleave GSDME to trigger pyroptosis in tumor cells [37, 75]. Moreover, neutrophil elastase, porcine epidemic diarrhea virus and Cathepsin G directly cleave GSDMD to induce pyroptosis [73, 74, 93]. SpeB also cleaves GSDMA and triggers pyroptosis [77]. However, these pathways have not been explored in ocular diseases. Matsubara et al. verified that GZMB is present in RPE and choroidal mast cells, and these pathways might become new research targets in ocular diseases [94].

## Pyroptosis in ocular diseases

### Retinal disease

**Fig. 3:**
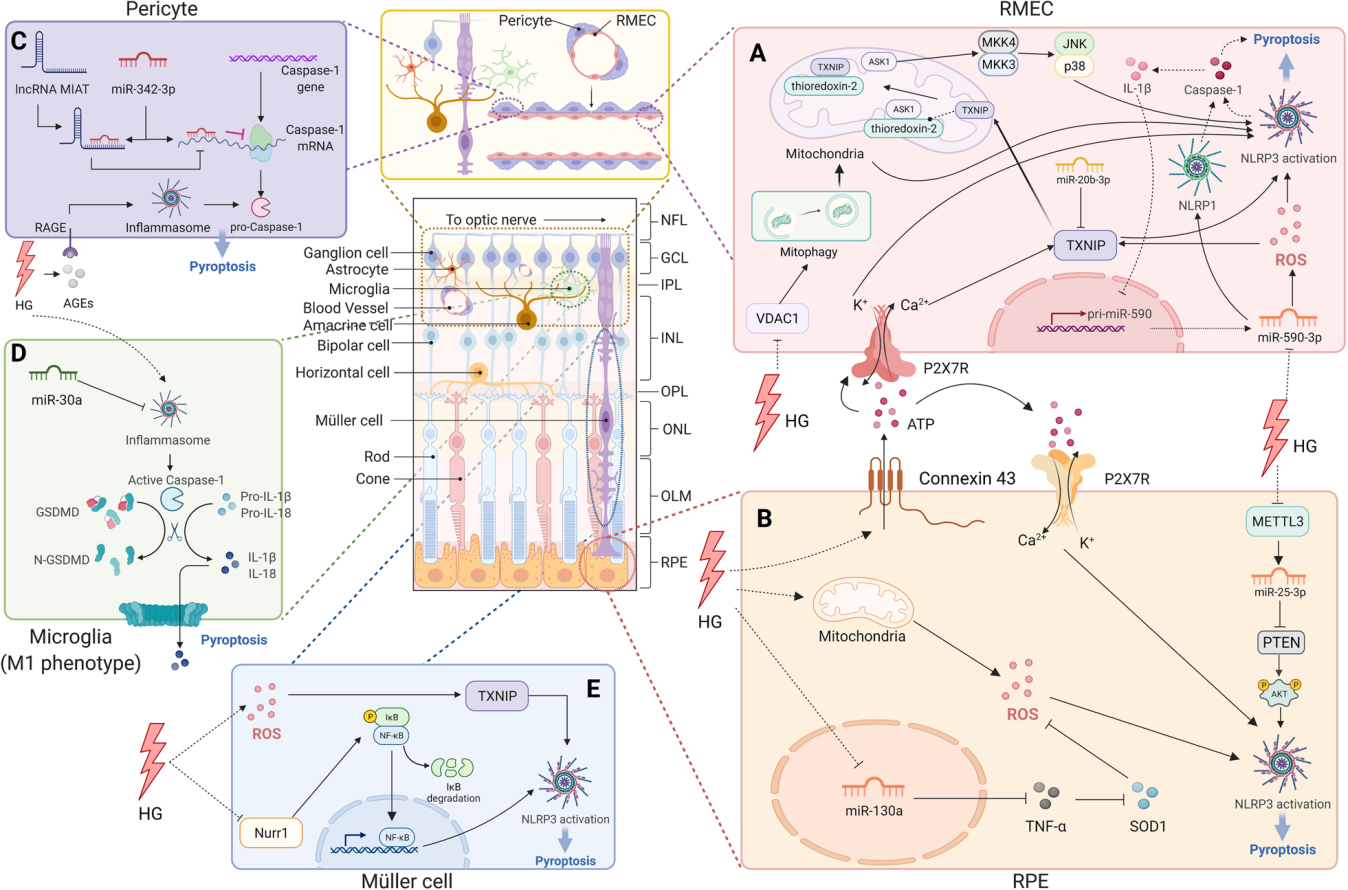
Molecular signaling pathway of pyroptosis in diabetic retinopathy. **A** Retinal microvascular endothelial cell (RMEC): increased extracellular ATP binds to P2X7R then activates NLRP3 inflammasome by causing K^+^ efflux and Ca^2+^ influx. TXNIP expression is induced through increased intracellular Ca^2+^ level, miR-590-3p/NOX4/ROS/TXNIP axis, or miR-20b-3p downregulation. After activation, the TXNIP shuttles to mitochondria, competes with apoptosis signal-regulating kinase 1 (ASK1), binds to NLRP3 and activates it; miR-590-3p also targets NLRP1. HG suppresses the voltage-dependent anion channel (VDAC1) expression, causing VDAC1/PINK1/Parkin-mediated mitophagy inhibition. Damaged mitochondria and mtROS accumulation results from impaired mitophagy-activated NLRP3 inflammasome. **B** Retinal pigment epithelium: hyperglycemia triggers the connexin43/ATP/P2X7R/Ca^2+^ influx pathway, mitochondrial ROS, miR-130a/TNF-α/SOD1/ROS pathway, or METTL3/miR-25-3p/PTEN/Akt/NLRP3 signaling cascade to activate NLRP3 inflammasome. **C** Pericyte: increased lncRNA MIAT competes with CASP1 mRNA for binding to miR-342-3p, blocking the CASP1 translation and CASP1-dependent pyroptosis. **D** Microglia (M1): MiR-30a downregulates NLRP3 expression. **E** Müller cell: hyperglycemia triggers the ROS/TXNIP axis to activate NLRP3 and downregulates the transcription factor nuclear receptor subfamily 4 group A member 2 (Nurr1) to suppress NLRP3 activation

**Fig. 4:**
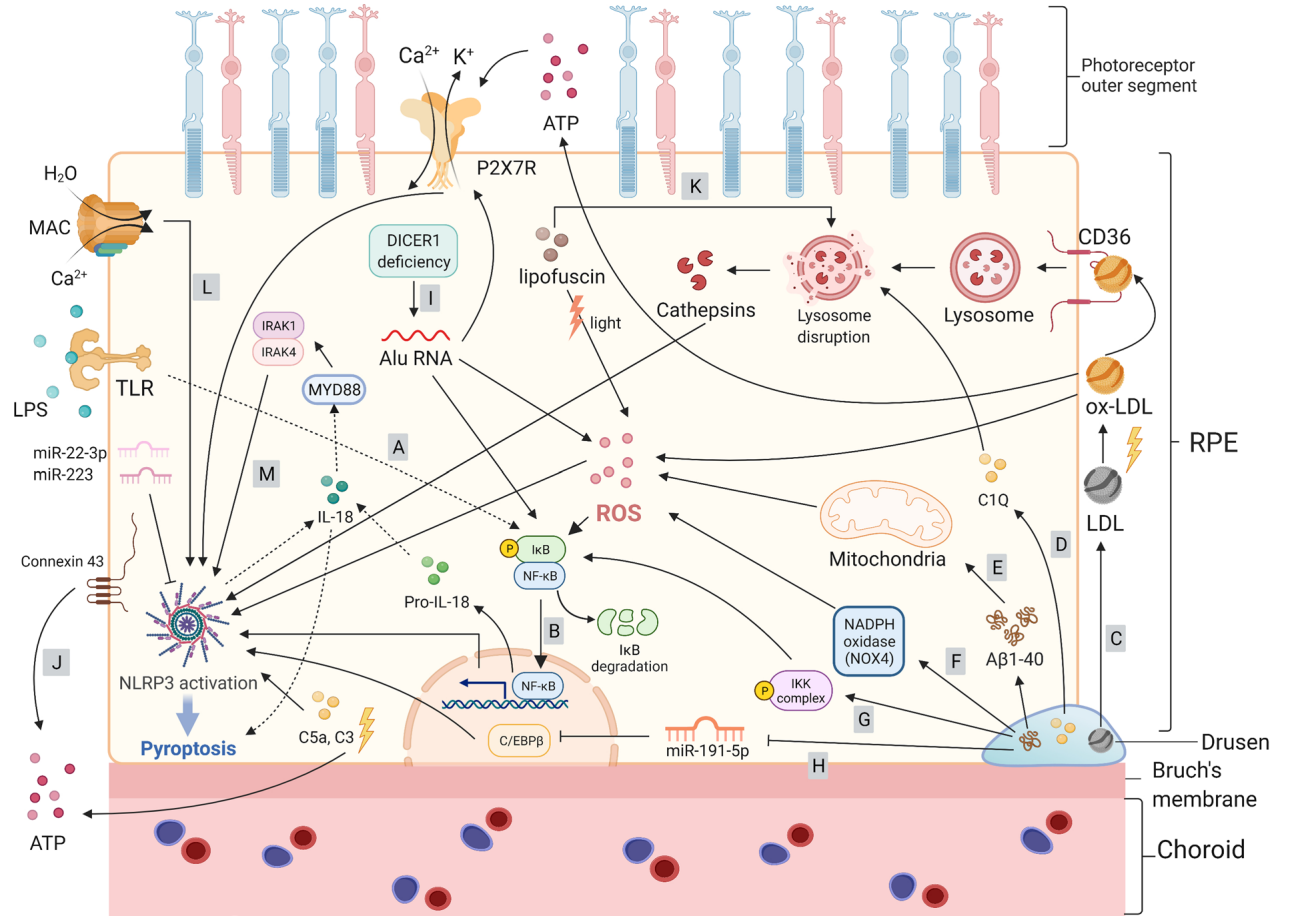
Molecular signaling pathway of pyroptosis in age-related macular degeneration. **A** Priming of NLRP3 with TLR. **B** Activation of the NF‑κB pathway induces the transcription of NLRP3 and pro‑IL‑1β. **C**–**H** Components of drusen activate the NLRP3 inflammasome. **C** Ox-LDL can induce a large amount of ROS, targeting the CD36 receptor, thereby causing lysosomal disruption following cathepsin release, or promoting the ATP/P2X7R/Ca^2+^ influx pathway to activate NLRP3. **D** Complement components: C1Q initiates lysosomal rupture and release of cathepsin B to activate NLRP3 assembly, while C5a and C3 send priming signals to NLRP3 without a clear mechanism. **E**, **F** Aβ1-40 increases intracellular ROS via NOX4 and mitochondrial electron transport chain to activate NLRP3. **G** Aβ1-40 acts as a priming signal to activate the NF-κB pathway, which upregulates the transcription of NLRP3, pro-IL-18, and pro-IL-1β. **H** MicroRNAs: miR-191-5p is downregulated after Aβ1-40 stimulation, subsequently leading to an increase in C/EBPβ levels, resulting in the upregulation of NLRP3; miR-223 and miR-22-3p suppress NLRP3 expression. **I** Alu RNA due to DICER1 deficiency increases ROS production; Alu RNA-induced NF-κB-mediated NLRP3 activation and P2X7R signaling control NLRP3 inflammasome priming and activation, respectively. **J** ATP outflow via connexin43 hemichannels acts as a NLRP3 inflammasome signal 2 activator. **K** A2E, a major fluorophore in lipofuscin, activates NLRP3 by causing lysosomal damage and release of cathepsins into the cytoplasm. **L** Membrane attack complex (MAC) deposition triggers the assembly and activation of the NLRP3 inflammasome downstream of the Aβ1-40 priming signal. **M** The mature form of IL-18 mediates the activation of interleukin-1 receptor-associated kinases 1 and 4 (IRAK1 and IRAK4), which contributes to RPE cell death

In recent years, with the increasing incidence of major chronic diseases such as diabetes and aging, the incidence of chronic blinding eye diseases such as DR and AMD is also gradually increasing, posing a new threat to human eye health. Therefore, the development of new diagnostic and therapeutic targets has become an important part of the research on ocular diseases. As the dynamic mechanism of disease can be elucidated at the cellular and molecular levels, it is possible to fundamentally solve the problem of cascaded pathological changes caused by changes at the molecular level and subsequent cell death. Pyroptosis, an important mode of cell death, is closely associated with the incidence and progression of retinal diseases. Retinal diseases, especially DR (Fig. 3) and AMD (Fig. 4), are the most well-studied pyroptosis-related ocular diseases. In addition, pyroptosis is also involved in retinal diseases of infectious pathogen origin, such as AIDS-related HCMV retinitis.

#### Diabetic retinopathy

DR is a common complication of diabetes mellitus and a major cause of vision loss and blindness in the working-age population worldwide [95]. Vascular abnormalities, blood–retinal barrier impairment, and neuronal dysfunction, which are caused by aberrant interactions among retinal, neuronal, glial, and vascular cells, are typical pathological changes in DR induced by long-term hyperglycemia [96]. Accumulating evidence shows that inflammasome activation facilitates DR [97]. As a more widely termed cell death form, inflammasome-related pyroptosis has been found to play a critical role in DR progression [17, 97]. Gan et al. showed that the hallmarks of pyroptosis, NLRP3-CASP1-GSDMD activation, membrane pore formation, and IL-1β and IL-18 are present in high glucose (HG)-induced human retinal pericytes [17]. Under HG conditions, pyroptosis is also triggered in microglia, human RMECs, and RPE cells [95, 98, 99].

Central in the process of pyroptosis, inflammasome activation induced by different stimuli is essential in DR pathogenesis and vision loss. A previous study demonstrated that DR progression was decelerated by inhibiting the NLRP3 inflammasome [100]. Hyperglycemia triggers increased expression and opening of connexin43 hemichannels on the cell membrane, which results in increased extracellular ATP [101]. Subsequently, increased extracellular ATP binds to P2X7R, a receptor that senses oxidative stress and metabolic stimulation that serves as an NLRP3 inflammasome activator, forming a nonselective cationic channel and causing the subsequent K^+^ efflux and Ca^2+^ influx, which directly activates the NLRP3 inflammasome, thereby triggering pyroptosis in human RMECs [101, 102]. Another study showed that glucose can pass through the plasma membrane and generate mitochondrial ROS. Consequently, the NLRP3 inflammasome is activated, causing CASP1 cleavage and IL‐1β and IL‐18 release, and resulting in pyroptosis in RPE cells [99]. There is also evidence showing that HG inhibits the expression of the voltage-dependent anion channel, a regulator of mitophagy anchored to the outer mitochondrial membrane, causing PINK1/Parkin-mediated mitophagy inhibition. Damaged mitochondria and mtROS accumulation resulting from impaired mitophagy activate the NLRP3 inflammasome in retinal capillary endothelial cells [103].

MicroRNAs have also been shown to be highly involved in the regulation of NLRP3. It has been demonstrated that miR-130a is remarkably downregulated under HG conditions in ARPE-19 cells and activates the TNF-α/SOD1/ROS signaling pathway to activate NLRP3-mediated pyroptosis [104]. In human RMECs incubated with the HG model, downregulation of miR-590-3p promoted pyroptosis through the NOX4/ROS/TXNIP/NLRP3 axis, as well as targeting NLRP1 [22]. In DR rats, researchers have shown that miR-20b-3p upregulation could confine the NLRP3-mediated inflammatory response by suppressing the thioredoxin-interacting protein (TXNIP), thereby retarding DR progression [105]. miR-30a downregulates NLRP3 expression by directly targeting its 3′-UTR [106]. In addition, miRNA modifications may play a role in pyroptosis. Research showed that overexpression of methyltransferase-like 3 (METTL3), which serves as a m^6^A modification “writer”, regulated miR-25-3p/PTEN/Akt/NLRP3 signaling in a DGCR8-dependent manner to alleviate HG-induced pyroptosis in RPE cell [13]. Furthermore, miRNAs can regulate pyroptosis by interacting with long non-coding RNA (lncRNA). In AGE-BSA-treated human retinal pericytes, the expression of lncRNA MIAT and CASP1 was strikingly increased, while miR-342-3p expression was decreased. Increased lncRNA MIAT may also compete with CASP1 mRNA for binding to miR-342-3p, resulting in the blockage of CASP1 translation as well as CASP1-dependent pyroptosis [107].

TXNIP is a widely overexpressed protein involved in cellular redox control in a high-glucose environment [105]. TXNIP also plays a prominent role in innate immunity via the TXNIP/NLRP3 inflammasome pathway and IL-1β secretion during DR development [97]. TXNIP acts as a bridge linking oxidative stress and the NLRP3 inflammasome in Müller cells during chronic hyperglycemia [108]. The dissociation of TXNIP from thioredoxin triggered by ROS transforms TXNIP from a thioredoxin repressor to an NLRP3 inflammasome activator [109]. Additionally, it was reported that HG induced TXNIP expression via increased intracellular Ca^2+^ levels or activation of carbohydrate response element-binding protein [110]. When activated, TXNIP shuttles to the mitochondria, where it competes for the binding site on thioredoxin-2 with apoptosis signal-regulating kinase 1 (ASK1). Thus, ASK1 is isolated from thioredoxin-2, thereby activating the following signaling pathway that contributes to NLRP3-related pyroptosis [95, 110].

Transcription factor nuclear receptor subfamily 4 group A member 2 (Nurr1) is a member of the superfamily of ligand-regulated transcription factors, the physiological ligands of which are unknown. It protects neurons from inflammation-induced cell death [111]. Nurr1 shows decreased expression and nuclear translocation in HG-treated Müller cells and exerts anti-inflammatory effects in astrocytes by binding to NF-κB p65 to prevent RGC loss [112]. It has been reported that protein kinase R regulates the NLRP3 inflammasome via NF-κB in RECs, which can be suppressed by Epac1 [113].

Multiple retinal cells are involved in DR due to the high-glucose environment, and evidence have demonstrated the crucial role of pyroptosis in pathogenesis. The chronic HG environment damages not only the retinal vascular units, but also other types of cells in the retina, such as RPE cells, microglia, and Müller cells, which are essential components of the blood–retinal barrier and key elements in maintaining the normal function of the visual system [114]. Extensive retinal cell death leads to the destruction of the blood–retinal barrier, which results in the leakage of liquid components from the blood vessels into the retina, causing bleeding, edema, exudation, and ischemia in the retina [115]. However, a previous study claimed that pyroptosis occurs prior to apoptosis [36], although this has not been proven in an ocular cell model. Although various ocular cell types suffer from pyroptosis in the pathogenesis of DR, the cell that is the initial victim remains unclear, which might become an important issue in the early diagnosis of DR, and the interaction among these cells within or after the pyroptosis process also requires further exploration. Moreover, the mode of cell death with the greatest effect on DR progression remains to be determined. In addition, studies on human samples are limited, and potential targets in mouse or cell models cannot be translated into clinical use in a short period of time.

#### Age-related macular degeneration

AMD is an aging problem and a leading cause of central vision loss and blindness [116]. Because of RPE damage, AMD is characterized by the degeneration of photoreceptors in the macula, which is a specialized area of the central retina that guarantees fine visual acuity [50]. Changes in RPE pigmentation, accumulation of lysosomal lipofuscin, and appearance of extracellular yellowish drusen deposits are the main characteristics of early-stage AMD [117]. Geographic atrophy (dry AMD) and choroidal neovascularization (wet AMD) are the two forms of late-stage AMD. The former is characterized by progressive and irreversible atrophy of the RPE, photoreceptors, and underlying choriocapillaris, whereas the latter is characterized by abnormal growth of choroidal vessels infiltrating the macula [50]. RPE cells, the monostromatic cells located between the Bruch’s membrane and the photoreceptors, are the main aggrieved cells in AMD [118]. In healthy retinas, RPE cells function as photoreceptor supporters by providing nutrients from the choroid and by removing waste products. They also maintain the integrity of the blood–retinal barrier and the stability of retinal pH, as well as play a part in the immune defense of the central retina. RPE cell degeneration is a consequence of complicated interactions between incremental inflammation and oxidative stress, mitochondrial dysfunction, and insufficient protein clearance [119]. Generally, when cells are attacked by an infection, pyroptosis induces pathological inflammation to accomplish its defensive role. Thus, it is worth noting that pyroptosis may take an important part in the progression of AMD. Moreover, it has been reported that pyroptosis could augment the susceptibility of RPE cells to photo-oxidative damage, leading to cell death and subsequent RPE degeneration [120].

Early research in this field mainly focused on inflammasomes, the central component of inflammation, of which the most widely studied one is NLRP3 [56]. NLRP3 is thought to contribute to AMD progression and has been extensively studied in AMD models. Drusen, one of the hallmarks of AMD, is located between the basal lamina of the RPE and the inner collagenous layer of the Bruch’s membrane and is believed to advance chronic inflammation in early stage AMD progression owing to its lipid and protein components [121]. It is thought to be involved in the pathogenesis of AMD by activating NLRP3 [122]. Drusen comprises various substances, such as Aβ, advanced glycation end products, complement components, oxidized lipids, and vitronectin [123]. Drusen is also enriched in low-density lipoproteins (LDL). Owing to its susceptibility to oxidation, LDL is easily transformed into ox-LDL, which induces a mass of ROS and oxidative stress [124]. In addition, ox-LDL can also be taken up by RPE by targeting the CD36 receptor and lysosomes, thereby causing their disruption. Lysosomal disruption, together with the release of cathepsins into the cytoplasm, results in NLRP3 activation and subsequent cascades of pyroptosis [124–126]. Studies have also shown that ox-LDL promotes ATP release, thereby activating P2X7R and resulting in Ca^2+^ influx and NLRP3 inflammasome activation [124].

Aβ, another important component of drusen, can also trigger NLRP3 inflammasome activation and stimulate NLRP3/GSDMD-dependent pyroptosis [10, 127]. Aβ exists in two forms: the 1–42 form in plaques of Alzheimer’s disease (AD) and the 1–40 form in AMD drusen [121]. Previous studies have confirmed that Aβ increases intracellular ROS via NADPH oxidase (NOX, especially NOX4) and the mitochondrial electron transport chain, which are the two main ROS sources in cells [121, 128]. Although the exact mechanism of ROS-induced NLRP3 inflammasome activation remains poorly understood, several studies have been conducted in this regard. Wang et al. revealed that the MAPK cascade activated by ROS initiated the translocation of NF-κB from the cytosol to the nucleus in LPS-primed ARPE-19 cells [121]. Aβ gives a priming signal to the NF-κB, which upregulates the transcription of NLRP3, pro-IL-18, and pro-IL-1β [129, 130]. The membrane attack complex on the RPE cell membrane provides a second signal for NLRP3 inflammasome assembly and activation [130]. Another study claimed that Aβ directly acts as a secondary activation signal after the priming step via the NF-κB pathway [129, 131].

Recently, miRNAs have been shown to be related to NLRP3 activation. For instance, after Aβ1-40 stimulation, miR-191-5p is downregulated in RPE cells, subsequently leading to an increase in C/EBPβ levels and resulting in increased transcription of NLRP3 [132]. Two other miRNAs, miR-223 and miR-22-3p, suppress NLRP3 expression [127, 133]. Complement components such as C1Q, C3a, and C5a are also involved in NLRP3 activation. Doyle et al. proposed that drusen component lysosomal rupture resulting from C1Q leads to the release of cathepsin B, a lysosomal exopeptidase, into the cytosol, which then activates NLRP3 assembly and activation [122]. Complement C5a also sends a priming signal for NLRP3 inflammasome activation and causes pyroptosis [134]. Notably, RPE cells can produce intracellular complement components (C3) under oxidative stress stimuli [135]. After treatment with properdin, scientists observed that the levels of C3 and NLRP3 were both downregulated. However, the underlying mechanism between C3 and NLRP3 activation remains unclear, and further investigation is required [135].

In addition to drusen on the Bruch’s membrane, the amount of insoluble lipofuscin, composed of nondegradable products of photoreceptor outer segment metabolism, accumulates with age within RPE cells [123]. A2E, a major fluorophore in lipofuscin, activates NLRP3 by disrupting lysosomes, thereby releasing cathepsins [123]. Other studies have shown that light-induced lipofuscin oxidation leads to the formation of lipofuscin oxidation products, causing excessive oxidative stress and ROS production, which directly activates the NLRP3 inflammasome and promotes the release of cytokines, causing further damage to RPE cells [134, 136, 137]. Risk factors that contribute to increased ROS (including mitochondrial ROS) generation and promote oxidative stress, such as aging, smoking, high-fat diet, light-induced photo-oxidative reactions, potassium efflux, and ATP, are also related to the activation of the NLRP3 inflammasome [56, 138–140]. Previous studies have reported that DICER1 deficiency results in an overload of Alu RNA, which also adds to excessive oxidative stress and ROS. ROS then primes the formation of the NLRP3 inflammasome and IL-18. Notably, the mature form of IL-18 is thought to mediate the activation of IL-1 receptor-associated kinases 1 and 4 (IRAK1/4), which is linked to NLRP3 activation and RPE pyroptosis [117, 141]. It has also been demonstrated that Alu RNA induces the NF-κB pathway and signaling via P2X7R to control the NLRP3 inflammasome priming and activation steps in the pathogenesis of geographic atrophy [142]. The release of ATP into the extracellular space via connexin43, which is overexpressed and opens channels prematurely in the RPE membrane, acts as a second signal of NLRP3 inflammasome activation [143, 144].

Although there is plenty of research in the field of AMD proving that NLRP3 inflammasome activation will lead to GSDMD-mediated pyroptosis in recent years [10, 124, 126, 127, 134, 145], most of the current research mainly focuses on the process of NLRP3 activation and lacks evidence revealing whether the RPE cell death form is indeed pyroptosis. Therefore, the mechanism between each stimulus and NLRP3/GSDMD pyroptosis remains to be elucidated. Furthermore, little is known about the role of other inflammasomes such as NLRC4 and AIM2 in the progression of AMD. It is worth noting that apart from NLRP3, noncanonical inflammasome activation (caspase-4/GSDMD-dependent pyroptosis) and caspase-3/GSDME-dependent pyroptosis have been shown to play an important role in the pathogenesis of AMD [69, 146]. Moreover, activated microglia, along with lysosomal destabilization, can act as a priming step to activate the NLRP3 inflammasome in RPE cells [147]. Therefore, the relationship between microglia and RPE pyroptosis is noteworthy.

According to the signaling pathways introduced above, the components of drusen are important triggers of RPE cell pyroptosis, which might accelerate the progression of AMD. Therefore, monitoring the early onset of drusen might be an effective diagnostic method for AMD, and blocking drusen-related molecules might be beneficial for slowing AMD progression. Notably, Aβ, one of the main components of drusen, also plays an important role in AD [148]. Hence, research targeting Aβ in AD might provide a clue on AMD therapy in the future.

#### AIDS-related HCMV retinitis

AIDS-related HCMV retinitis is a sight-threatening retinal disease caused by beta-herpesvirus that affects AIDS patients who do not have access to antiretroviral therapy or who fail to respond to it, as well as non-AIDS patients who are immunosuppressed for solid-organ or bone marrow transplantation [149, 150]. In order to investigate the pathogenesis of AIDS-related HCMV retinitis, scientists have conducted works with a well-characterized model of experimental murine cytomegalovirus (MCMV) retinitis in mice with retrovirus-induced immunosuppression (MAIDS) [149]. Apoptosis was previously thought to be the major contributor to the retinal pathology that develops after the onset of AIDS-related HCMV retinitis [151]. However, Chien et al. challenged this assumption by showing that only 4% of retinal cells were undergoing apoptosis in MCMV-infected eyes of MAIDS-10 mice (mice with MAIDS of 10-week duration) while molecules related to pyroptosis and necroptosis significantly increased [150]. For further investigation, this team analyzed and compared the intraocular expressions of immune response genes within MCMV-infected eyes of groups of healthy mice and MCMV-infected mice with MAIDS via transcriptional analysis [152]. The significant intraocular stimulation of pyroptosis-associated genes, such as CASP1, IL-1β, IL-18, and AIM2 inflammasome, served to confirm their previous determination [152]. Xu et al. also provided evidence that the expression of pyroptosis-related inflammasomes, including NLRP3, NLRP4, NLRP6, and AIM2 were upregulated in the eyecups of the MCMV-infected mice [153]. Of greater significance, Carter et al. recently demonstrated that although both pyroptosis and apoptosis occur in the ocular compartment of MCMV-infected mice with MAIDS, pyroptosis contributes far more than apoptosis toward the development of full-thickness retinal necrosis during the pathogenesis of MAIDS-related MCMV retinitis [14]. An atypical phenomenon was also observed in this research that the MCMV-infected eyes of MAIDS mice with caspase-1, GSDMD, or IL-18 deficiency did not develop full-thickness retinal necrosis but instead presented thickening and proliferation of the retinal pigmented epithelium layer with relative preservation of the neurosensory retina in contrast to wildtype MAIDS mice [14]. Above all indicate that pyroptosis might be an operative target for MAIDS-related MCMV retinitis. More investigation of this model and its future application in AIDS patients is worth expecting.

##### Glaucoma

**Fig. 5:**
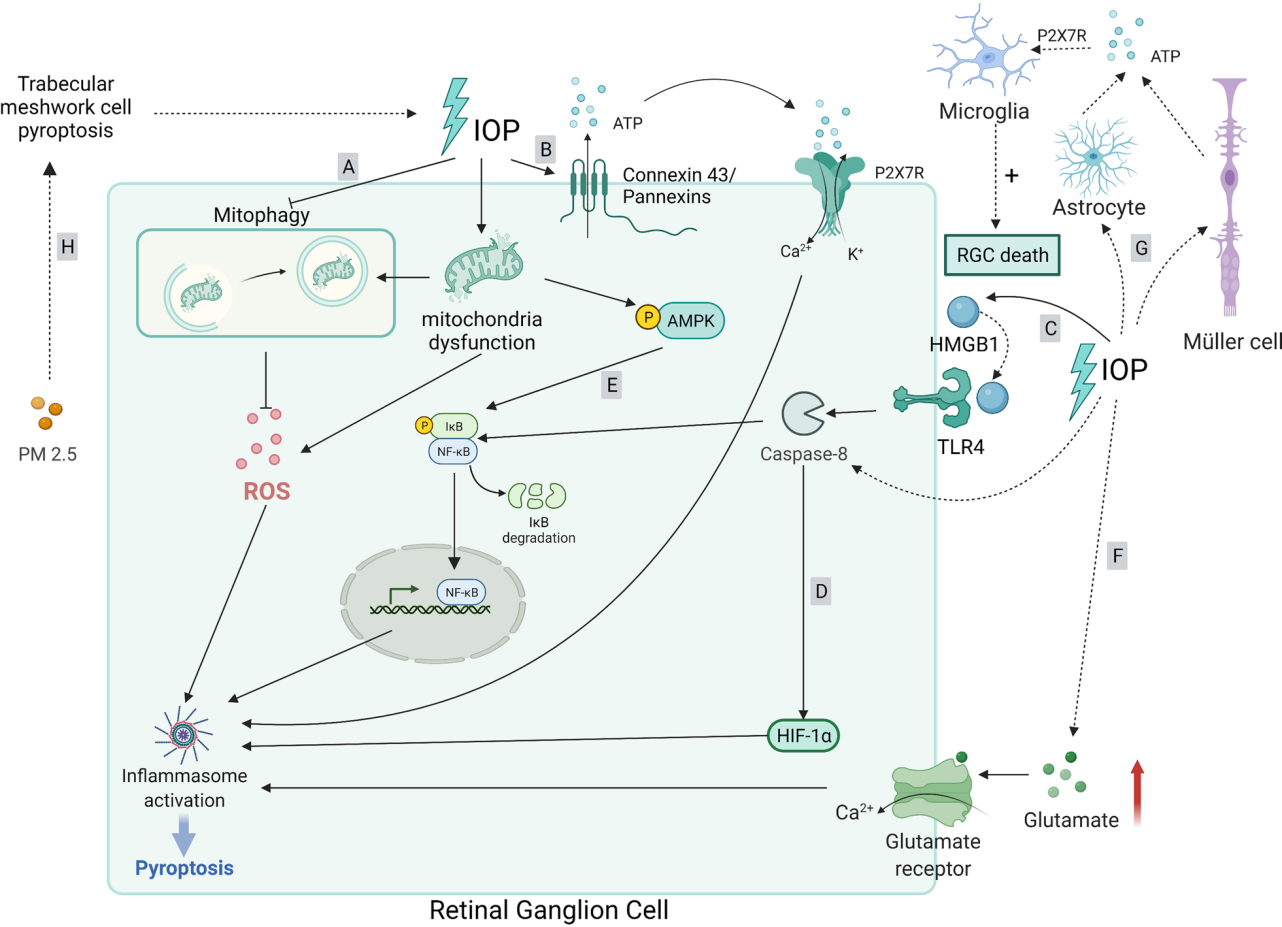
Molecular signaling pathway of pyroptosis in glaucoma. **A** Elevated IOP results in the inhibition of hypoxia-induced mitophagy. As dysfunctional and fragmented mitochondria accumulate, the subsequent oxidative stress promotion induces NLRP3 activation. **B** Elevated IOP triggers pannexin and connexin hemichannels, induces ATP efflux, and promotes the ATP/P2X7R/Ca^2+^ influx pathway to activate the NLRP1/NLRP3/AIM2 inflammasome. **C** Retinal ischemic reperfusion injury triggers the release of high-mobility group box 1 (HMGB1) in the retina, which binds to TLR4, promotes the activation of caspase-8, subsequently regulating the activation of NLRP3 via NF-κB pathway. **D** CASP8-HIF-1α signaling is an upstream regulator of NLRP3/NLRP12/NLRC4 in high IOP-induced retinal ischemic injury and may initiate pyroptosis. **E** AMP-activated protein kinase (AMPK) activates NF-κB signaling and induces NLRP3 inflammasome assembly. **F** Elevated glutamate binds to the glutamate receptors on RGCs, causing a large influx of Ca^2+^ and mitochondrial dysfunction, thus triggering NLRP3 inflammasome activation. **G** Ocular hypertension drives astrocytes or Müller cells to release ATP to activate microglial cells via the ATP/P2X7R/NLRP3 pathway, which contributes to RGC death. **H** PM2.5, triggers oxidative stress, which activates NLRP3-mediated pyroptosis in trabecular meshwork cells and results in ocular hypertension

Glaucoma is a neurodegenerative disease that can eventually lead to irreversible blindness [154]. Glaucoma causes damage to the optic nerve and progressive degeneration of RGCs [62]. IOP, older age, family history, and non-White race are essential risk factors for this complex multifactorial disease [155]. IOP increases as a result of blocked aqueous humor outflow from the eye. This reduces the amount of blood flow to the eye, causing hypoxia or ischemia. Furthermore, mechanical damage, oxidative stress, and hypoxia induce mitochondrial dysfunction in RGCs, glial activation, and neuroinflammation, magnifying the pathogenesis of glaucoma [156]. Chronic neuroinflammation not only directly damages RGCs, but also establishes a pro-inflammatory environment and impairs the immune privilege of the retina [156]. Specifically, the NLRP3 inflammasome, a key driver of pyroptosis and inflammation, has been demonstrated to engage in glaucoma pathogenesis (Fig. 5).

NLRP3 inflammasome activation has been reported in pre-clinical studies of glaucoma. Pro-inflammatory cytokines resulting from NLRP3 inflammasome activation are increased in the blood of patients with glaucoma, resulting in the death of RGCs due to neurotoxic inflammation [157]. IOP elevation activates NLRP3 in the glial cells of the retina, leading to neurotoxic inflammation, axon degeneration, and subsequent death of RGCs in glaucoma [157]. In a physiological state, hypoxia caused by elevated IOP induces mitophagy to reduce the number of dysfunctional mitochondria. However, this process may be inhibited in patients with glaucoma. Accumulation of damaged mitochondria promotes oxidative stress and metabolic vulnerability. Oxidative stress then activates NLRP3, resulting in further inflammatory response [154]. Concomitantly, elevated IOP triggers pannexin and connexin hemichannels, inducing ATP efflux from the intracellular space [23, 156]. External ATP binds to and opens P2X7R, resulting in the uptake of extracellular Ca^2+^ [23]. The Ca^2+^ influx then signals the assembly of the NLRP1/NLRP3/AIM2 inflammasome, mediating GSDMD pore formation to execute pyroptosis [23]. Nevertheless, each type of inflammasome is activated in a different cellular pattern. In particular, NLRP1 is active in RGCs, NLRP3 is poorly active in astrocytes and vasculature but is inducible in ganglion cell layer neurons, and AIM2 is active in Müller glia [23]. Moreover, Ca^2+^ influx can induce inflammation, microglial activation, and cytokine release by stimulating mitochondrial destabilization [154]. Retinal ischemic reperfusion injury triggers the release of HMGB1 in the retina, which is an endogenous ligand of TLR4, and activates caspase-8 and the downstream NF-κB pathway to initiate NLRP3 activation [64, 158].

Additionally, activated caspase-8/NF-κB can trigger hypoxia-inducible factor-1α (HIF-1α) signaling [11, 159]. CASP8-HIF-1α signaling regulates NLRP3/NLRP12/NLRC4 in retinal ischemic injury induced by high IOP, and this axis may initiate CASP1/GSDMD-mediated pyroptosis. Furthermore, scientists found that IL-1β magnifies pyroptosis by promoting the aforementioned axis, revealing that IL-1β exacerbates neuroinflammation and pyroptosis [11]. Metabolic vulnerability also contributes to inflammation in glaucoma. The limited energy supply in the optic nerve and retina triggers the primary cellular energy sensor, AMP-activated protein kinase, which activates NF-κB/NLRP3 signaling [159]. Furthermore, glutamate excitotoxicity is assumed to be a major factor in the onset and progression of glaucoma [160]. In glaucoma, there is a relatively high level of glutamate in the extracellular fluid [161]. Excessive glutamate interacts with the glutamate receptors on RGCs, resulting in a large Ca^2+^ influx and mitochondrial dysfunction, thus triggering NLRP3 inflammasome activation that leads to RGC death [162]. Apart from the NLRP3 inflammasome, studies have shown that NLRC4 is also involved in RGC pyroptosis in an acute glaucoma mouse model, but the precise mechanism requires further investigation [163].

The retina has a self-defense system that consists of three types of resident glial cells (astrocytes, Müller cells, and microglia) that mediate the inflammatory response [157, 164]. Glial cells play a key role in determining the life-or-death decisions of neurons in glaucoma by exerting both supportive and detrimental effects. Various stress stimuli create an imbalanced environment in the glaucoma tissue, which initiates and propagates secondary damage (neuroinflammation). Chronic activation of glial cells is generally regarded as a sign of ongoing neuroinflammation in the glaucomatous retina and optic nerve [165]. Evidence has shown that ocular hypertension drives astrocytes or Müller cells to release ATP, and microglia induced by the ATP/P2X7R/NLRP3 pathway contribute to RGC death [166]. However, the role of pyroptosis in this process remains to be elucidated. Notably, pyroptosis appears to link environmental factors, such as fine particulate matter (PM2.5), to the onset of glaucoma. PM2.5 triggers oxidative stress, which activates NLRP3-mediated pyroptosis in human trabecular meshwork cells. Thus, IOP is elevated due to blocked aqueous humor outflow, which initiates the progression of ocular hypertension and glaucoma [18].

In conclusion, IOP elevation initiates multiple signaling pathways of pyroptosis and leads to RGC death. As a result, targeting these related molecules may slow down or even reverse RGC degeneration. Additionally, the exact role of resident glial cells associated with pyroptosis of RGCs is a potential issue that needs to be determined. Furthermore, previous studies have mainly focused on signaling after the onset of IOP elevation; however, only a few studies have focused on the causes of increased IOP. For example, environmental factors might elevate IOP by triggering pyroptosis of trabecular meshwork cells and blocking the outflow of aqueous humor. Therefore, more questions regarding the etiology of glaucoma need to be answered.

##### Ocular surface disease

The ocular surface is directly and constantly exposed to environmental irritants, allergens, and pathogens. Therefore, it loads a first-line self-defense system, a rapid immune response, to maintain its integrity [167]. Corneal and conjunctival epithelial cells are now classified as innate immunity cells, which are traditionally leukocytes and serve as not only a bridge between innate and adaptive immunity, but also as a first-line defense against pathogens [168]. Ocular surface diseases have one thing in common as they involve direct changes in the chemistry of the ocular surface as well as the properties of the tear film and the topographical properties of cellular components or the microbial distribution of the ocular surface. These diverse diseases should find a way to identify the underlying etiological factors and develop effective treatment strategies through interdisciplinary research [169]. In recent years, emerging studies have found that pyroptosis is a newly discovered mode of ocular surface cell death and engages in the pathogenesis of ocular surface disorders such as DED and keratitis.

###### Dry eye disease

DED is a common ocular disease that is characterized by tear film insufficiency. Without proper intervention, the main symptom of ‘eye irritation’ may aggravate and progress to visual disturbances [170]. Tear hyperosmosis, corneal inflammation and injury, corneal neuroparesthesia, and dysfunction of the lacrimal and meibomian glands all contribute to DED pathogenesis [171]. Of these, inflammation is considered a key factor in DED and is regarded as both the cause and outcome of DED [172]. Serving as the outermost layer of the eye, corneal and conjunctival epithelium frequently confront large amounts of environmental factors, such as low humidity, air pollution, air conditioning, and longer viewing digital screen hours, which increase tear evaporation, decrease tear volume, lead to tear hyperosmosis, cause aridity stress, and induce ROS production, thus triggering the death of corneal and conjunctival epithelium [168, 173].

Excessive generation of ROS and oxidative stress in DED contribute to inflammation [172]. Zheng et al. first showed that increased ROS leads to NLRP3 inflammasome activation and CASP1-mediated IL-1β secretion in a murine DE model [174]. By analyzing tear samples and conjunctival specimens from patients with DED, researchers found upregulated expression of NLRP3 inflammasome and downstream CASP1, IL-1β, and IL-18, especially in patients with Sjögren’s syndrome (SS) dry eye [175]. Subsequently, another study reported that NLRP3 mediated innate inflammation after ROS generation in hyperosmotically stressed human CECs. These studies suggest that the ROS/NLRP3/IL-1β signaling pathway may function as a priming step in DED development [176]. Chi et al. demonstrated that ROS overproduction under hyperosmolarity causes mitochondrial DNA-oxidized damage, which may stimulate BRCC36, a DNA damage-associated deubiquitinase, to activate the NLRP3 inflammasome while suppressing the NLRP6 inflammasome [168]. This study also proved that tear hyperosmosis activated NLRP3 and noncanonical IL-1β processing via CASP8 activation [168]. A study also showed that inhibiting hyperosmolarity-induced inflammation through mitigation of the translocation of NF-κB to the nucleus decreases NLRP3-mediated inflammatory response, suggesting that ROS activates NLRP3 protein expression via NF-κB signaling [173]. A more recent study showed that excessive ROS can promote the formation of NLPR3 by dissociating TXNIP from thioredoxin [172]. Furthermore, Zhang et al. showed that NLRP3/CASP1/GSDMD-driven pyroptosis is involved in hyperosmosis-induced CEC damage [177].

In addition to NLRP3, scientists have shown that TLR4-induced CASP8 activation facilitates the collaboration between NLRP12 and NLRC4 inflammasomes, triggering GSDMD-dependent pyroptosis in the mucosal corneal epithelium in response to aridity stress [12]. Moreover, this signaling can be magnified by IL-33, thereby forming a cascade of inflammation in DED [12].

Overall, the studies mentioned above have shown that NLRP3 is now the most well-studied inflammasome in DED, and ROS and excessive oxidative stress are the major triggers of NLRP3 activation. Future research should focus on other inflammasome types. A recent study on SS revealed a marked elevation of the AIM2 inflammasome and increased spontaneous IL-1β production. However, the team used only salivary biopsy tissues and ductal salivary gland epithelial cell lines in patients with SS [178]. However, it provides a new prospect for the study of pyroptosis in SS dry eye such that the AIM2 inflammasome may also participate in the pathogenesis of DED. Analysis of the lacrimal gland and tear samples from patients with SS may be the next step for future research on SS.

###### Keratitis

Fungal keratitis is a corneal disease caused by fungal infection that may lead to blindness [19]. CECs are responsible for the first-line immune response against fungi. After recognizing PAMPs through PRRs, a series of signal transduction pathways, such as NF-κB, is activated in CECs, which elevates the expression of IL-1β [179]. *Aspergillus fumigatus* keratitis is one of the most well-studied fungal keratitis, and emerging studies have shown that pyroptosis plays a role in its pathogenesis. Zhao et al. concluded that interferon (IFN) stimulated by *A. fumigatus* in the cornea binds to homologous IFNR, causing JAK/STAT signaling transduction and triggering caspase-1/GSDMD-mediated pyroptosis [19]. Another study showed that *A. fumigatus*-stimulated CECs induce pyroptosis of THP-1 macrophages by secreting thymic stromal lymphopoietin, a type of inflammatory factor produced by epithelial cells [34]. Yang et al. demonstrated that *A. fumigatus* infection leads to the opening of pannexin 1 channels, which then activates NLRP3/CASP1/IL-β pathway [180]. In addition to *A. fumigatus*, *Pseudomonas aeruginosa* is also an essential pathogen for keratitis. In corneal samples from patients with *P. aeruginosa* keratitis and rat models, CASP4/5/11 expression was upregulated. LPS-induced noncanonical pyroptosis occurs in this model [181]. Qu et al. showed that triggering receptors expressed on myeloid cells 2, a PRR expressed on myeloid cells, promotes host defense against *P. aeruginosa* keratitis by inhibiting CASP1-dependent pyroptosis [182]. Previous studies have shown that *P. aeruginosa* infection promotes the assembly of NLRC4 and NLRC3 inflammasomes [183, 184].

Apart from fungi, herpes simplex virus type 1 (HSV-1) is also an infectious pathogen that leads to cornea inflammatory disease. Early during viral infections, NLRP3, NLRP12, and IFI16 inflammasomes are activated, leading to elevated production of biologically active CASP1, IL-1β and IL-18 along with an increased recruitment of monocytes and neutrophils into the inflamed cornea, which then results in clinical inflammation that exacerbates corneal manifestation [185]. Another study also reported that NLRP3 activation was induced by HSV-1 infection in the cornea of both murine and human CECs [186]. The initial purpose of inflammasome activation might serve as a protective strategy for host to fight against viral immunopathological lesion [187]. Nevertheless, dysregulation or hyper-activation of different inflammasomes by virulent strains of HSV-1 can make the ‘protective’ inflammatory responses pass the point of no return, and finally lead to an exacerbation of inflammation that is harmful to the well-being of the cornea [185]. Therefore, controlling the type and the level of activation of the host’s inflammasomes might become a potential target for preventing further progression of HSV-1-related keratitis caused by aberrant inflammation.

In other keratopathies such as diabetic corneal endothelial keratopathy, lncRNA KCNQ1OT1 sponges miR-214, which targets CASP1 as a competing endogenous RNA (ceRNA) and regulates the expression of IL-1β during pyroptosis [188]. Niu et al. demonstrated that PM2.5, the chief culprit of air pollution, induces the ROS/NLRP3/GSDMD-mediated pyroptosis axis in human CECs [189]. In corneal alkali burn, the TLR4–MyD88–CASP8 axis causes imbalance in NLRP3/NLRP6 (upregulation of NLRP3 and downregulation of NLRP6) and subsequent corneal epithelial damage [190].

##### Uveitis

Uveitis is a complex inflammatory ocular disease that involves uvea, the middle layer of the eye between the retina and the sclera. Uveitis can be classified across several dimensions. According to the anatomic location of inflammation, uveitis is classified into anterior uveitis (anterior chamber), intermediate uveitis (vitreous cavity), posterior uveitis (retina, choroid, retinal blood vessels), and panuveitis (anterior chamber, vitreous, and retina or choroid with no one site predominant) [191]. Etiologically, uveitis can be classified into infectious (antigen mediated), non-infectious (immune mediated), or masquerade (cancer associated) [15, 191]. Non-infectious uveitis is often shown as a manifestation of autoimmune or inflammatory systemic diseases, including juvenile arthritis, Vogt–Koyanagi–Harada disease, sarcoidosis, Blau syndrome, and Behcet’s disease, etc. [15, 192]. Both innate and adaptive immune system are engaged in the inflammatory pathways underlying uveitis. The innate immune system plays a major role in infectious uveitis, while the non-infectious uveitis largely depends on the adaptive immune system [193]. The innate immune system is the first-line host defense against microbial invasion and relies on PRRs, such as NLRs and TLRs. When PRRs recognize danger signals (PAMPs or DAMPs), innate immune cells or inflammasomes are activated, resulting in the recruitment of leukocytes at the site of infection or extracellular release of cytokines, which then promotes a cascade of inflammatory response to eliminate the invading pathogen [15, 194, 195]. The adaptive immune system includes cell-mediated (T cell) and humoral-mediated (B cell) immunities, both of which are key to drive tissue inflammation or repair [195]. CD4+ and CD8+ T cells, as well as antibodies, are adaptive immune components that can express effector function in the eye [196]. Naïve CD4+ T cells are able to be differentiated into diverse subsets of effector T cells, such as T-helper (Th) 1, Th2, and Th17, when they are triggered by antigen peptide stimulation and specific cytokines [15, 195]. From data in different animal models of uveitis, both Th1 and Th17 cells specific to retina are induced [197]. Cytokines released by Th1 (e.g., IL-1, IL-18, IL-6, etc.) and Th17 (e.g., IL-17, IL-21, IL-22, etc.) will aggravate lymphocyte infiltration and ocular inflammation, leading to an inflammatory microenvironment in the eye by facilitating recruitment of neutrophils, macrophages, and monocytes into the eye, as well as promoting Th cell differentiation, clonal expansion, and cytokine production [15, 198, 199]. IL-1β, a cytokine that is secreted by Th1 and plays a critical in both pyroptosis and Th17 cell differentiation, must be processed into its mature form under the strict regulation of inflammasomes [198]. IL-18, another important cytokine in pyroptosis, has great effect on modulating both innate and adaptive immune system [199]. Therefore, understanding the interaction underlying Th1, Th17, cytokines and inflammasomes will be necessary to figure out the role of innate and adaptive immune system in the pathogenesis of uveitis and will add to our further investigation on pyroptosis in the pathogenesis of uveitis.

As an essential part of immune system, although there is very little direct morphological evidence, pyroptosis is thought to be related to the pathogenesis of uveitis for its key players showing great activities in the pathogenesis of uveitis. Up to now, a number of inflammasomes have been demonstrated to be involved in the pathogenesis of uveitis. Li et al. demonstrated that NLR signaling pathway is one of the most prominent pathways in the iris tissues in experimental autoimmune uveitis [200]. A study identified that NLRP1, NLRP3, and NLRC4 inflammasomes might be associated with posterior segment uveitis susceptibility using whole-exome sequencing [201]. NLRP12 has recently been implicated in regulation of inflammation and protection against experimental autoimmune uveitis (EAU) [202]. NLRP12−/− mice developed exacerbated uveitis, which was not attributed to inherent T cell dysfunction but depended on enhanced neutrophil and macrophage accumulation, indicating that NLRP12 exerts an anti-inflammatory role to suppress uveitis [202]. In peripheral blood mononuclear cell samples of patients with Behçet’s disease, which is one of the uveitis-related chronic inflammatory diseases, NLRP3 expression level is upregulated during the onset of disease [203]. Genetic variants of NLRP3 and NLRP1 gene were found in patients suffering Behçet’s disease. And the autoinflammatory syndromes of these patients were thought to be caused by a misregulation of the inflammasome proteins that leads to an increased release of IL-1β [204–207]. Apart from Behçet’s disease, cryopyrin-associated periodic syndrome, a group of inherited autoinflammatory disorders caused by NLRP3 mutations, shows ocular symptoms, including anterior uveitis [208]. Rosenzweig et al. verified that although NLRP3 and CASP1 were activated and responsible for controlling the production of IL-1β in endotoxin-induced uveitis model, deficiency in NLRP3 or CASP1 did not significantly alter the severity of uveitis [209]. Another study in EAU model also confirmed this point. NLRP3 inflammasome and the following production of IL-1β are elevated in EAU mice. In contrast, NLRP3 inflammasome were not elevated in complement-deficiency mice. However, EAU-associated retinal pathophysiology were not improved in complement-deficiency mice [210]. These surprising findings cast doubt on the role of NLRP3 inflammasome in the pathogenesis of uveitis, raising the question of whether it is necessary or dispensable for the development of uveitis. Hence, more investigations on a more specific role of inflammasomes in uveitis and their involvement with other immune response elements, such as T cells, are required in the future.

Functional CASP1 formed by inflammasome complex cleaves pro-IL-1β and pro-IL-18 into bioactive IL-1β and IL-18. IL-1 signaling is pivotal for the pathogenesis of uveitis. Early in 1994, Yoshida et al. observed elevated expression level of IL-1β in the iris–ciliary body of endotoxin-induced uveitis model [211]. Mice that lack IL-1 receptor show decreased inflammation in the model of uveitis [212]. Moreover, the eye of mice that is deficient in IL-1 receptor antagonist (Ra), which is an endogenous inhibitor of IL-1 activity, shows extreme sensitivity to LPS and marked exacerbation of the resulting uveitis [213]. A study also found that lentivirus gene delivery of IL-1Ra significantly reduced the severity of experimental uveitis, implying that lentivirus-mediated expression of immunomodulatory genes in the anterior chamber could be used to treat uveitis [214]. Zhao et al. presented that the severity of inflammation was correlated with expression level of IL-1β, IL-1Ra, and IL-18 in the aqueous humor of patients with HLA-B27-associated acute anterior uveitis [215]. In addition, Fabiani et al. observed that the symptoms of uveitis in patients with Behçet’s disease were suppressed after receiving anti-IL-1 agents, indicating the vital significance of IL-1 signaling in the development of uveitis. However, a research showed that mice with deficiency in IL-1Ra shows low susceptibility to NOD-2-mediated uveitis, which belongs to the manifestation of Blau syndrome [216]. Therefore, the difference of susceptibility to uveitis across patients with different inflammatory disorders and the involvement of IL-1 signaling in the severity of uveitis are pending further research. Although there has been research showing an elevation of IL-18 in aqueous humor of patients with uveitis [215, 217], and an increased susceptibility to uveitis in patients with Behçet’s disease along with IL-18 promoter polymorphisms [218], it is a pity that strategies targeting IL-18 signaling have not drawn much attention from scientists focusing on uveitis.

To the best of our knowledge, there is only very limited direct evidence of pyroptosis in research on uveitis. Despite this, there are reasons to believe that pyroptosis may play a role in the pathogenesis of uveitis and be related to the exacerbating ocular inflammation during uveitis, based on the vibrant activities of key players of pyroptosis, such as inflammasomes and cytokines, in the pathogenesis of uveitis, as well as recent evidence showing induced pyroptosis in intestinal epithelial cells derived from patients with Behçet’s disease [219].

##### Cataracts

Cataracts, characterized by lens opacity, are the most common ocular disease and the leading cause of blindness worldwide [20]. Drugs, malnutrition, aging, ultraviolet (UV) light, and diabetes mellitus are widely accepted risk factors for cataract [220]. LECs can stabilize the intracellular environment and maintain a clear crystalline lens [221]. Oxidative damage occurs when LECs are exposed to both endogenous and exogenous oxidative stress, including light radiation and inflammatory factors, producing high levels of ROS. ROS overproduction has been reported to induce NLRP3/CASP1 activation, which subsequently triggers IL-1β/IL-18 production and cell death by pyroptosis [221]. The present study demonstrated that pyroptosis may participate in cataract formation in a model of H_2_O_2_-treated LECs via CASP1/IL-1β pathway [221]. Sun et al. showed that CRTAC1-mediated UVB-induced pyroptosis in human LECs occurs via ROS/NLRP3/CASP1 signaling [20]. Other studies have shown that short-wavelength blue light induces pyroptosis by activating the canonical or noncanonical CASP1/GSDMD signaling axis in LECs [220, 222]. In addition, the expression levels of CASP1 and GSDMD in LECs changed with blue light exposure times [222].

## Potential therapies targeting pyroptosis for ocular diseases

The role of pyroptosis in the initiation and progression of ocular diseases has drawn the attention of scientists seeking inhibitors or agents that specifically target pyroptosis pathway-related proteins, such as NLRP3, CASP1, GSDMD, and other molecules (Table 2).

**Table 2:** Summary of the potential agents of pyroptosis in ocular diseases

Disease	Agent	Target	Pathway	Promote (+)/suppress (−) ODs	Animal/cell model	References	Research in other fields
Diabetic retinopathy	Vitamin D3	ROS	ROS/TXNIP/NLRP3	−	Patient vitreous sample/human retinal microvascular endothelial cells	[109, 246]	Acute kidney injury/human tubular epithelial cell [247] Hepatic injury [248]
	Ghrelin	ROS	ROS/NLRP3	−	ARPE-19/Wistar rats	[115]	Nonalcoholic fatty liver disease [249] Multiple sclerosis/neuroinflammation [250] Traumatic brain injury-induced acute lung injury [251]
	Hydrogen sulfide (H2S)	ROS	ROS/NLRP3	−	ARPE-19	[114]	Ischemia–reperfusion (I/R)-induced acute kidney injury [252] Diabetic cardiomyopathy [253, 254]
	MCC950	NLRP3	TXNIP/Nek7-NLRP3/caspase-1/GSDMD	−	Retinal microglia, C57BL/6J mice, human retinal endothelial cells	[98, 100, 255]	Aβ accumulation [256]
	β-Hydroxybutyrate	NLRP3	ER stress/NLRP3	−	C57BL/6J mice	[229]	Renal ischemia/reperfusion injury [233]
	Methylene blue (MB)	NLRP3	NLRP3/caspase-1	−	Sprague–Dawley rats	[230]	Myelodysplasia [234]
	Z-YVAD-FMK	caspase-1	Caspase-1/IL-1β	−	Retinal microglia	[98]	Lung inflammation [257] Neuroblastoma [258]
	H3 relaxin	P2X7R	P2X7R/NLRP3	−	Human retinal microvascular endothelial cells	[102]	Nephrocalcinosis [259]
	METTL3	miR-25-3p	miR-25-3p/PTEN/Akt	−	ARPE-19	[13]	Diabetic kidney disease [260] Liver fibrosis (+) [261]
	lncRNA MIAT	miR-342-3p	miR-342-3p/caspase-1	+	Human retinal pericyte	[107]	Diabetic cardiomyopathy [262]
Age-related macular degeneration	A740003 (P2X7 receptor antagonist)	P2X7R	P2X7R/ROS/NLRP3	−	ARPE-19 cells C57BL/6 mice	[124]	Osteoarthritis [232]
	miR‐22‐3p	NLRP3	NLRP3/caspase-1	−	ARPE‐19/Balb/c mice	[133]	Cerebral ischemia/reperfusion injury [263]
	INF39	NLRP3	NLRP3/caspase-1	−	ARPE‐19	[126]	Liver injury [264] *Mycoplasma pneumoniae* infection [265]
	Tranilast	NLRP3	NLRP3/caspase-1	−	ARPE‐19	[227]	Atherosclerosis [266]
	*Lycium barbarum* polysaccharides (LBP)	amyloid β1-40 (Aβ1-40)	Aβ1-40/NLRP3/caspase-1/GSDMD	−	ARPE-19	[10]	–
	Baicalin	miR-223	miR-223/NLRP3/caspase-1	−	ARPE-19	[127]	Hepatic injury in non-alcoholic steatohepatitis [267]
Glaucoma	Melatonin	NF-κB	NF-κB/NLRP3/caspase-1/GSDMD	−	Sprague–Dawley rats/ganglion cell	[223]	Atherosclerosis [268] Hepatic ischemia/reperfusion injury [269] Cerebral ischemia [270] Diabetes-induced brain injury [271]
	Kaempferol	NF-κB	NF-κB/NLRP3/caspase-1	−	Retinal ischemia–reperfusion (I/R) mice model	[62]	Spinal cord injury [235] Parkinson’s disease [236]
Dry eye disease	Calcitriol	NLRP3	NLRP3/caspase-1/GSDMD	−	Human corneal epithelial cells	[177, 272]	–
	Disulfiram	GSDMD	GSDMD	−	Human corneal epithelial cells	[177]	Severe acute pancreatitis [273] Lipopolysaccharide-induced sepsis/ulcerative colitis [274]
Keratitis	Wedelolactone	IKK	Caspase-4/5/11/GSDMD	−	Human corneal keratocytes	[181]	Acute pancreatitis [245]
	Disulfiram	GSDMD	GSDMD	−	Neutrophil and macrophage	[241]	Severe acute pancreatitis [273] Lipopolysaccharide-induced sepsis/ulcerative colitis [274]
Diabetic corneal endothelial keratopathy	lncRNA KCNQ1OT1	miR-214	miR-214/caspase-1	+	Corneal endothelial	[188]	Diabetic cardiomyopathy [275] Diabetic nephropathy [276]
Uveitis	Anakinra	IL-1β	IL-1β/IL-1R	−	Patients with Behçet’s disease	[277]	Atherosclerosis [278] Muscle inflammation [279] Acute liver failure [280]
	Canakinumab	IL-1β	IL-1β/IL-1R	−	Patients with Behçet’s disease	[277, 281]	Myocardial infarction [282]
	Minocycline	TLR4	TLR4/IL-1β	−	Rat endotoxin-induced uveitis model	[283]	Depression [284] Spinal cord injury [285]

*MIAT* myocardial infarction associated transcript, *ROS* reactive oxygen species, *NLRP* NOD-like receptor protein, *GSDMD* gasdermin D, *IKK* inhibitor of nuclear factor kappa-B kinase, *TXNIP* thioredoxin interacting protein, *PTEN* phosphatase and tensin homolog, *ARPE-19* adult retinal pigment epithelial cell line-19, *IL-1R* interleukin-1 receptor, *ODs* ocular diseases

Several studies have provided explicit evidence that agents such as MCC950, H3 relaxin, melatonin, INF39 and calcitriol can alleviate ocular cell pyroptosis by directly suppressing NLRP3 [98, 102, 126, 177, 223]. Among which, MCC950 and INF39 are well-accepted NLRP3 inhibitor for the function of blocking NLRP3 ATP hydrolysis motif and inhibiting the interaction of Nek7-NLRP3, respectively [224–226]. Tranilast, another direct NLRP3 inhibitor which suppresses NLRP3 assembly by blocking NLRP3 oligomerization, shows an inhibitory effect on RPE cell death by a combination of mitochondrial antioxidant and other anti-inflammatory drugs [227]. Notably, it has been reported that β-carotene, a plant-derived pro-vitamin A that prevents the progression of multiple eye diseases, is capable of blocking NLRP3 by directly binding to the NLRP3 PYD [228]. However, whether it can prevent pyroptosis effectively in ocular models remains unclear. Hence, it has the potential to be an effective agent for pyroptotic ocular cell death. Other NLRP3 inflammasome inhibitors have also been identified to be involved in slowing down the progression of ocular diseases, such as β-hydroxybutyrate, methylene blue, sulforaphane, A740003, and kaempferol [62, 124, 229–231]. Although these studies only focused on inhibiting the activation of NLRP3 and lack evidence on pyroptopic cell death in ocular tissues, scientists in other fields support the potential therapeutic effect of these agents on pyroptosis in other diseases, such as β-hydroxybutyrate on renal ischemia/reperfusion injury, methylene blue on myelodysplasia, A740003 on osteoarthritis, and kaempferol on spinal cord injury and Parkinson’s disease [232–236]. These agents can suppress ATPase activity, block the P2X7 channel, inhibit the NF-κB signaling pathway, prevent NLRP3 oligomerization, or disturb ATP binding to the NACHT domain. Apart from the aforementioned agents, there are other agents or molecules that indirectly obstruct NLRP3 activation. Vitamin D3 decreases ROS levels and further suppresses the ROS/TXNIP/NLRP3 inflammasome pathway in HG-induced RMECs [108]. Ghrelin and hydrogen sulfide have been reported to reduce the inflammatory response and pyroptosis by blocking the ROS/NLRP3/CASP1 signaling pathway in HG-induced RPE [114, 115]. Baicalin increases the expression of miR-223, thus suppressing NLRP3-triggered pyroptosis and alleviating AMD progression [127]. Unfortunately, these agents are only used in cells or animal models and cannot be used in clinical applications. For instance, although the well-known NLRP3 inhibitor MCC950 exhibits great potency and high target selectivity, its pharmacokinetic and toxicokinetic properties restrict its therapeutic use in the clinic [237]. Therefore, their clinical application in patients with ocular diseases remains a long way to go.

CASP1 inhibitors are also agents that can inhibit pyroptosis in ocular tissues, such as VX-765 and Z-YVAD-FMK [18, 19, 98]. VX-765 acts by covalent modification of the catalytic cysteine residue in the active site of caspase-1 [238]. In addition to the canonical pathway, wedelolactone has therapeutic potential for targeting CASP4/5/11 in *P. aeruginosa* keratitis [181]. The interaction between lncRNAs and miRNAs may also become a new therapeutic target for pyroptosis in ocular diseases. Overexpression of the lncRNA MIAT alleviated CASP1 repression by sponging miR-342-3p, thereby promoting CASP1-mediated retinal pericyte pyroptosis. The MIAT/miR-342-3p/CASP1 axis may offer new insights for the development of therapies for DR [107]. In addition, lncRNA KCNQ1OT1 acts as a ceRNA that competitively binds miR-214 to regulate caspase-1 and promote diabetic corneal endothelium dysfunction, which might offer new biomarkers or new targets for treatment [188].

Blocking the executor GSDMD might also be useful in interfering with pyroptosis in ocular diseases. Cellular FLICE inhibitory protein (c-FLIP), an anti-apoptotic regulator similar to CASP8, can cleave GSDMD and cause neuronal pyroptosis in the retina [239]. Therefore, inhibition of c-FLIP may provide a new approach to mitigate retinal neuron pyroptosis. In the DED model, the GSDMD blocker disulfiram decreased the number of cells undergoing pyroptosis and helped cells fight HS-induced cytotoxicity by covalently modifying human/mouse Cys191/Cys192 in GSDMD thereby blocking pore formation [177, 240]. More recently, a study revealed that disulfiram, together with antifungal agents, may become a new treatment for reducing corneal opacity in fungal keratitis [241].

In addition, targeting IL-1 or IL-18 signaling is an effective and possible strategy to control ocular inflammation in autoinflammatory diseases, such as uveitis [15, 199]. Anakinra, canakinumab and minocycline are reported to reduce ocular inflammation in uveitis model via intervening IL-1 signaling [242]. Moreover, gene therapy for pyroptosis-related gene variants might contribute to advanced treatment for non-infectious uveitis in the future [243, 244].

The aforementioned agents showed only a small fraction of potential agents. Although there were more potential agents that were related to pyroptotic proteins in other studies, we only selected the agents (Table 2) that were also proven to be involved in pyroptosis in non-ocular diseases, such as renal diseases, neuronal diseases, cardiovascular diseases, and hepatic diseases. Therefore, multidisciplinary studies might provide new insights into potential therapies for ocular diseases, and scientists should look for common ground in the pathogenesis of different diseases and seek effective agents to mitigate pyroptosis. In addition, many studies have shown that ocular diseases may involve different modes of cell death. Apart from determining the exact relationship between all kinds of cell death modes and how much they are individually involved in the pathogenesis of each disease, we also need to look at agents that could target different cell death modes at the same time or find agents that target the intersection points of the intricate signaling pathway (e.g., wedelolactone [245]). Nevertheless, we must be aware that some of the known agents have limited potential in clinical use because of their pharmacokinetic and toxicokinetic properties, for example, MCC950 [237]. Therefore, searching for their isoforms with lower toxicity to humans and subsequent pharmaceutical synthesis is definitely another mountain that needs to be conquered.

## Conclusion and prospect

With increasing research, pyroptosis, an essential mode of PCD, has been gradually uncovered. In this review, we compared the characteristics of pyroptosis with other types of cell death and summarized the mechanisms and pathways of pyroptosis, as well as the key players of pyroptosis such as inflammasomes and gasdermins. Pyroptosis is a type of lytic cell death characterized by swift plasma membrane disruption, followed by leakage of cellular contents and cytokines (IL‐1β and IL‐18). It is executed by gasdermins, which function as pore-forming proteins on the cell membrane and can be activated in an inflammasome-dependent (CASP1-dependent pathway and CASP4/5/11-dependent pathway) or-independent (caspase-3/8-mediated, other GSDM-mediated) manner. The studies highlighted in this review present exciting new evidence that pyroptosis plays a prominent role in various ocular diseases such as DR, AMD, AIDS-related HCMV retinitis, glaucoma, DED, uveitis, cataract, and keratitis.

Previously, the inflammasome-dependent pathway of pyroptosis was generally accepted by researchers. However, emerging evidence has shown that inflammasome-independent pathways also play a crucial role in cell death and disease progression. However, few studies have been conducted on the latter pathway in ocular disease. During the exploration of different types of cell death, we found that the relationship between cell death forms is complicated, and myriad ties between pyroptosis and other cell death in ocular diseases remain to be discovered. At the same time, this is also a common problem in current research on pyroptosis or other types of cell death. In disease models, one death approach is often studied separately, whereas in general, the death method of cells is a dynamic change under the pathological conditions of the body. Most likely, the coexistence of multiple modes of death, and even until now, the links between the modes of cell death in many disease progressions have not been clearly elucidated. In follow-up scientific research, it is worth noting that the process of cell injury and death should be focused on and studied as a whole. In addition to proving the existence of pyroptosis or other specific death modes in diseases, more attention should be paid to the dynamic link between modalities, such as pyroptosis and other deaths. The dynamic relationship between the modalities will be more meaningful for translation and application in clinical treatment. Furthermore, there are still many unresolved issues at the molecular level of pyroptosis. For example, the activation of inflammasomes, caspases, and other proteins does not necessarily cause pyroptosis. The role of each member of the GSDM family in different diseases, which is the ultimate executor of pyroptosis, as well as the difference between the GSDM family's pore punching and MLKL-induced pore-breaking in necroptosis, all require the development of new identification methods and a more comprehensive understanding to accurately distinguish between the different types of cell death.

Targeting NLRP3/CASP1/GSDM, which are the central molecules in pyroptosis, may become a novel strategy to interfere with the occurrence or progression of ocular diseases. Currently, the quantities of inhibitors and agents targeting pyroptosis-related proteins have shown therapeutic potential in pre-clinical studies of ocular diseases, including NLRP3 inhibitors (MCC950, H3 relaxin, melatonin, and calcitriol), caspase inhibitors (VX-765 and Z-YVAD-FMK), and the GSDMD inhibitor disulfiram. In addition, IL-1 signaling intervention and gene therapy targeting pyroptosis-associated gene variants also draw much attention from scientists in order to control ocular inflammation effectively. Although we summarize some of the inhibitory agents or potential molecular targets of pyroptosis in ocular diseases, most of the evidence regarding inflammasome and pyroptosis activation in ocular disorders comes from in vitro and experimental models in cells or animals, and effective clinical trials of pyroptosis-targeted agents have not been conducted in ocular fields. We cannot accurately determine whether the aforementioned NLRP3/CASP1/GSDMD agonists or inhibitors can be used to treat ocular diseases in the human body. When it comes to the authentic clinical effectiveness of a drug, the combination of other drugs and pyroptosis inhibitors can also be considered to achieve better therapeutic effects. In addition, gene therapy and nanodrugs (i.e., drugs encapsulated with nanoparticles) targeting the pyroptosis pathway will also have satisfactory application prospects and are some of the research directions that are expected to achieve clinical breakthroughs in the future. We hope that basic research on pyroptosis and ocular diseases will continuously improve and translate into clinical practice quickly, safely, and successfully.

## Data Availability

Not applicable.

## Abbreviations

AD: Alzheimer’s disease
AIM2: Absent in melanoma-2
ALRs: Absent in melanoma-2-like receptors
AMD: Age-related macular degeneration
Apaf-1: Apoptotic protease activating factor 1
ASC: Apoptosis-associated speck-like protein containing a caspase activation and recruitment domain
ASK1: Apoptosis signal-regulating kinase 1
atRAL: All-trans retinal
Aβ: Amyloid-beta
Bcl-2: B-cell lymphoma 2
CARD: Caspase activation and recruitment domain
CASP: Caspase
CECs: Corneal epithelial cells
ceRNA: Competing endogenous RNA
c-FLIP: Cellular FLICE inhibitory protein
cyt c: Cytochrome c
DAMPs: Damage-associated molecular patterns
DED: Dry eye disease
DR: Diabetic retinopathy
dsDNA: Double-stranded DNA
EAU: Experimental autoimmune uveitis
FADD: Fas-associated protein with death domain
GSDMD: Gasdermin D
GZMs: Granzymes
HCMV: AIDS-related human cytomegalovirus
HMGB1: High mobility group box 1
HSV-1: Herpes simplex virus type 1
IOP: Intraocular pressure
LDL: Low-density lipoproteins
LECs: Lens epithelial cells
lncRNA: Long non-coding RNA
LPS: Lipopolysaccharide
LRR: Leucine-rich repeat
MAIDS: Retrovirus-induced immunosuppression
MCMV: Murine cytomegalovirus
METTL3: Methyltransferase-like 3
MOMP: Mitochondrial outer membrane pore
MyD88: Myeloid differentiation primary response 88
NACHT: Nucleotide-binding domain
NAIPs: Neuronal apoptosis inhibitory proteins
Nek7: NIMA-related kinase 7
NF-κB: Nuclear factor-κB
NLRP3: NOD-like receptor protein 3
NLRs: Nucleotide-binding oligomerization domain- and leucine-rich repeat-containing receptors
NOD: Nucleotide-binding oligomerization domain
NOX: NADPH oxidase
Nurr1: Nuclear receptor subfamily 4 group A member 2
PAMPs: Pathogen-associated molecular patterns
PCD: Programmed cell death
PFD: Pore-forming domain
PM2.5: Fine particulate matter
PRRs: Pattern recognition receptors
PYD: PYRIN domain
Ra: Receptor antagonist
RGCs: Retinal ganglion cells
RMECs: Retinal microvascular endothelial cells
SpeB: Streptococcal pyrogenic exotoxin B
SS: Sjögren’s syndrome
T3SS: Type III secretion systems
TAK1: TGF-β-activated kinase-1
Th: T-helper cells
TRIM: Tripartite motif
TXNIP: Thioredoxin-interacting protein
UV: Ultraviolet
